# Chinese visceral adiposity index and its transition patterns: impact on cardiovascular and cerebrovascular diseases in a national cohort study

**DOI:** 10.1186/s12944-024-02105-0

**Published:** 2024-04-29

**Authors:** Zhihan Lv, Yunxi Ji, Su Xu, Chenyi Li, Wenwei Cai

**Affiliations:** 1 Department of General Medicine, Shanghai Ninth People’s Hospital, Shanghai Jiao Tong University School of Medicine, No. 639 Zhizaoju Road, Shanghai, 200011 China; 2 Department of Endocrine and Metabolic Diseases, Shanghai Institute of Endocrine and Metabolic Diseases, Ruijin Hospital, Shanghai Jiao Tong University School of Medicine, Shanghai, China

**Keywords:** Chinese visceral adiposity index, Visceral adipose tissue, Cardiovascular and cerebrovascular diseases, Longitudinal transition

## Abstract

**Background:**

Obesity affects approximately 800 million people worldwide and may contribute to various diseases, especially cardiovascular and cerebrovascular conditions. Fat distribution and content represent two related yet distinct axes determining the impact of adipose tissue on health. Unlike traditional fat measurement indices, which often overlook fat distribution, the Chinese visceral adiposity index (CVAI) is a novel metric used to assess visceral fat accumulation and associated health risks. Our objective is to evaluate its association with the risk of cardiovascular and cerebrovascular diseases.

**Methods:**

A nationwide longitudinal study spanning 9 years was conducted to investigate both the effects of baseline CVAI levels (classified as low and high) and dynamic changes in CVAI over time, including maintenance of low CVAI, transition from low to high, transition from high to low, and maintenance of high CVAI. Continuous scales (restricted cubic spline curves) and categorical scales (Kaplan-Meier curves and multivariable Cox regression analyses) were utilized to evaluate the relationship between CVAI and cardiovascular and cerebrovascular diseases. Furthermore, subgroup analyses were conducted to investigate potential variations.

**Results:**

Totally 1761 individuals (22.82%) experienced primary outcomes among 7717 participants. In the fully adjusted model, for each standard deviation increase in CVAI, there was a significant increase in the risk of primary outcomes [1.20 (95%CI: 1.14–1.27)], particularly pronounced in the high CVAI group [1.38 (95%CI: 1.25–1.54)] compared to low CVAI group. Regarding transition patterns, individuals who consistently maintained high CVAI demonstrated the highest risk ratio compared to those who consistently maintained low CVAI [1.51 (95%CI: 1.31–1.74)], followed by individuals transitioning from low to high CVAI [1.22 (95% CI: 1.01–1.47)]. Analysis of restricted cubic spline curves indicated a positive dose-response relationship between CVAI and risk of primary outcomes (p for non-linear = 0.596). Subgroup analyses results suggest that middle-aged individuals with high CVAI face a notably greater risk of cardiovascular and cerebrovascular diseases in contrast to elderly individuals [1.75 (95% CI: 1.53–1.99)].

**Conclusion:**

This study validates a significant association between baseline levels of CVAI and its dynamic changes with the risk of cardiovascular and cerebrovascular diseases. Vigilant monitoring and effective management of CVAI significantly contribute to early prevention and risk stratification of cardiovascular and cerebrovascular diseases.

**Supplementary Information:**

The online version contains supplementary material available at 10.1186/s12944-024-02105-0.

## Introduction

Since 1975, the global prevalence of obesity among adults has almost tripled [1]. In the United States, approximately 42% of adults are affected by obesity, resulting in an estimated annual healthcare cost of $173 billion [2, 3]. Presently, China stands as the country with the highest number of individuals affected by overweight and obesity worldwide [4], with an estimated 789.95 million by 2030 [5, 6]. According to the WHO’s definition [7], Obesity and overweight are characterized by the excessive or abnormal accumulation of fat, which is associated with significant health risks, particularly cardiovascular and cerebrovascular diseases [8–10].

Visceral adipose tissue (VAT) constitutes a critical component of fat distribution and serves as an independent predictor for various pathophysiological conditions. When assessing an individual’s obesity level and studying cardiovascular and cerebrovascular diseases, the distribution of fat holds greater significance than its mere quantity [11]. Over recent decades, epidemiological studies have indicated that visceral adipose tissue may stand as an independent risk marker for the onset and mortality associated with cardio-cerebrovascular diseases [12, 13], notably among the elderly [14].

Currently, methods for evaluating VAT include anthropometric measures, bioelectrical impedance analysis (BIA), and imaging indicators, each carrying distinct advantages and limitations [15]. Anthropometric indicators, including body mass index (BMI) and waist circumference (WC), function as pivotal indicators for assessing population-level overweight or obesity in adults. BMI stands as a crucial gauge of an individual’s overall body mass status. However, its limitations are evident as BMI variations exist significantly within the same individual’s body composition, incapable of distinguishing between subcutaneous and visceral fat [16], potentially leading to the “obesity paradox“ [17, 18]. Evidently, BMI fails to account for individual heterogeneity in fat distribution associated with increased risk of cardiovascular disease. BIA can also estimate VAT but yields indirect outcomes based on fat-free tissue measurements, lacking precision and accuracy. With significant advancements in medical imaging, precise measurement of VAT is achievable through CT, MRI, or DEXA [19]. However, these methods may involve radiation exposure or economic constraints, limiting their widespread clinical implementation [20, 21].

The visceral adiposity index (VAI) and lipid accumulation product (LAP) reflect the accumulation of VAT and identify dysfunction within visceral fat, with multiple studies demonstrating its association with cardiovascular metabolic disorders [22, 23]. However, these metrics were primarily established using data from Western populations, which inherently makes them less tailored for the Chinese demographic. Notably, the Chinese visceral adiposity index (CVAI), proposed by Xia [24], integrates anthropometric and biochemical variables, tailored to the characteristics of the Chinese population. Previous studies have demonstrated that, compared to conventional indices, CVAI holds superior clinical value in conditions like hypertension [25–27], diabetes [28–30], fatty liver [31, 32], and carotid artery plaque [33, 34]. Presently, there is a paucity of research regarding the association between CVAI and cardiovascular and cerebrovascular diseases, highlighting the imperative need for further validation of their relationship.

To fill this evidence gap, a nationwide study was undertaken, utilizing a comprehensive analysis of longitudinal data spanning from 2011 to 2020. The primary objective was to elucidate the association between CVAI and cardiovascular and cerebrovascular diseases among the elderly population in China.

## Materials and methods

### Study design and participants

This study involved participants sourced from the China Health and Retirement Longitudinal Study (CHARLS, data accessible at http://charls.pku.edu.cn), which is dedicated to the compilation of a high-caliber data repository in China. An in-depth description of the CHARLS’s design has already been published [35–37]. In general, CHARLS is a substantial, prospective, nationwide, observational cohort that specializes in middle-aged and elderly people. To ensure the representativeness of the study population, it employs a multi-stage stratified probability proportionate sampling method, surveying approximately 19,000 individuals across more than 12,000 households, with follow-up surveys conducted at intervals of 2 to 3 years [38, 39]. Besides, blood samples were also collected from participants in the 2011 and 2015 surveys. Ethical approval was obtained from Peking University’s Institutional Review Board for all study waves, and participants were required to provide written informed consent before taking part in the study.

**Fig. 1:**
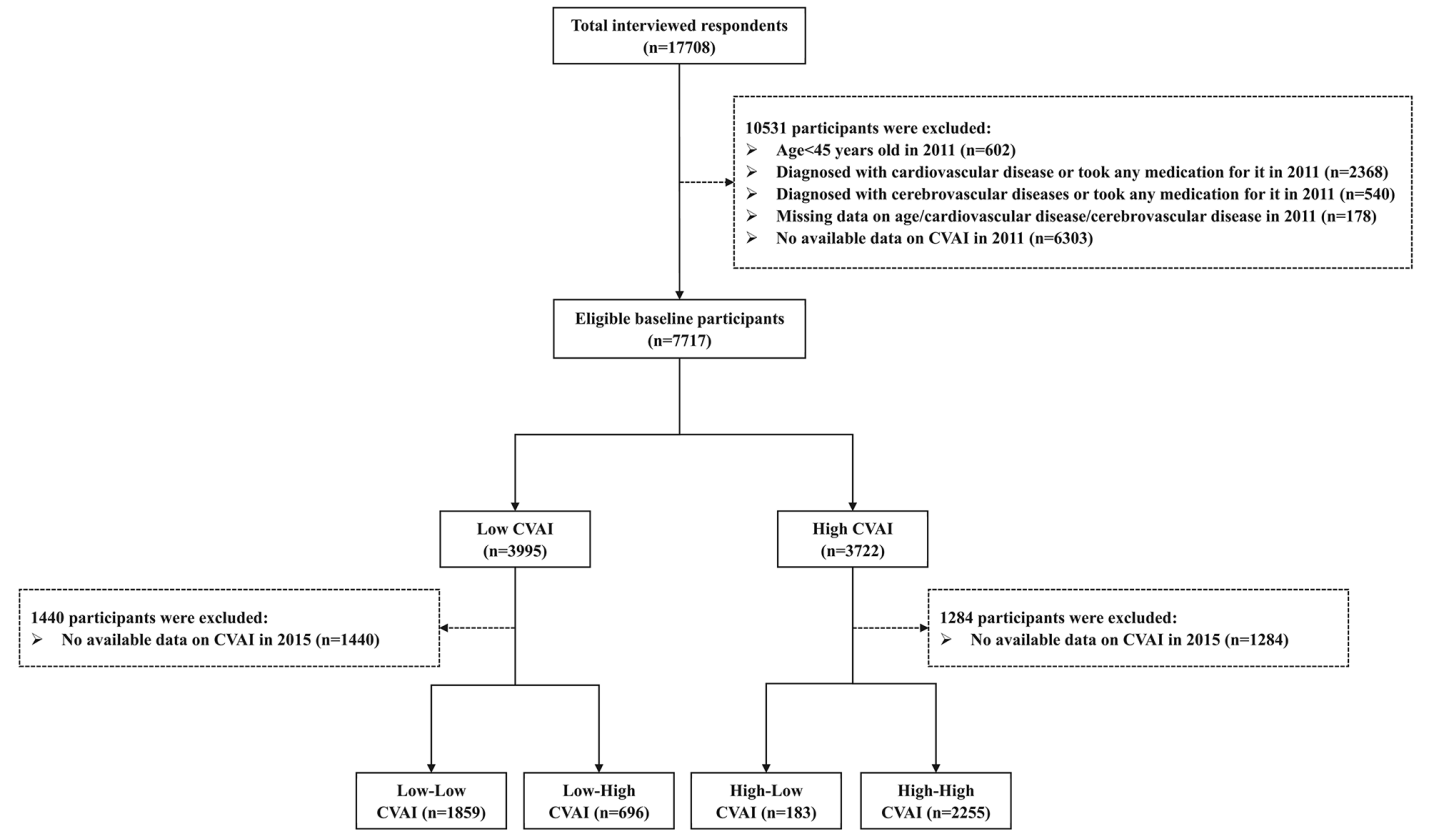
Flowchart for subjects included in this study. High group: CVAI ≥ 88.42 (females), ≥ 101.80 (males); Low group: CVAI < 88.42 (females), < 101.80 (males)

Participants aged 45 years and older, free from a documented history of cardiovascular and cerebrovascular disease, met the criteria for inclusion in the study. Participants with incomplete records regarding their cardiovascular and cerebrovascular disease history were excluded from the analysis. Furthermore, individuals with insufficient data to calculate the CVAI were also excluded from the analysis. Details regarding the specific criteria for inclusion and exclusion can be found in Fig. 1.

### Data collection and measurement

The longitudinal dataset obtained from CHARLS includes a wide range of variables such as demographics, concomitant comorbidities, health behaviors, socioeconomic status, physical measurements, and biochemical variables. Trained investigators administered a structured questionnaire through a Computer-Assisted Personal Interviewing (CAPI) system to collect this information [36]. Anthropometric indicators were measured following standardized protocols and guidelines. Uniform instruments (Seca^TM^213 stadiometer and Omron^TM^HN-286 scale) were used for height (cm) and weight (kg) measurements, respectively, and documented to one decimal point. During WC measurements, participants were directed to maintain calm breathing, exhale completely, and hold their breath after exhaling. A soft measuring tape was then positioned horizontally at the level of the navel for accurate measurement [36].

For the collection of biochemical variables, participants were required to fast overnight and underwent blood draws the following morning by trained professionals. After verifying participant identities, the blood samples were carefully labeled to ensure accurate matching. Within 2 hours of collection, all blood samples were promptly delivered to a local laboratory for comprehensive blood count analysis. Subsequently, using an extensive cold-chain shipping network for biological specimen transportation, samples were directly dispatched to the central laboratory network at the research headquarters. At this central location, standardized and widely acknowledged methods were employed to measure additional indicators such as blood glucose (hexokinase), lipid profile (oxidase method and direct method), glycated hemoglobin (high-performance liquid chromatography), and renal function (enzymatic and picric acid method). The central laboratory is accredited with the ISO 15189 certification from the College of American Pathologists and the International Organization. Quality control specimens are run daily by the laboratory, with the CHARLS team providing weekly oversight of the results [40].

### Exposure

CVAI is a simplified index established based on anthropometric and biochemical variables to evaluate visceral fat distribution among the Chinese demographic. Prior research has demonstrated CVAI’s effectiveness as a reliable measure of abdominal obesity within the Chinese population [24, 41]. We compared BMI, WC, LAP, VAI, and CVAI in predicting cardiovascular and cerebrovascular diseases among middle-aged and elderly individuals in China. Details of the calculation formulas for each indicator are presented in Table S1. For CVAI, we applied the widely recognized gender-specific calculation formula as follows:

$$\begin{array}{l}Male: - 267.93 + 0.68 \times age\left( y \right) + 0.03 \times BMI\left( {kg/{m^2}} \right) + \\4.00 \times WC\left( {cm} \right) + 22.00 \times LgTG\left( {mmol/L} \right) - \\16.32 \times HDL\left( {mmol/L} \right)\end{array}$$

$$\begin{array}{l}Female: - 187.32 + 1.71 \times age\left( y \right) + 4.23 \times BMI\left( {kg/{m^2}} \right) + \\1.12 \times WC\left( {cm} \right) + 39.76 \times \\LgTG\left( {mmol/L} \right) - 11.66 \times HDL\left( {mmol/L} \right)\end{array}$$

The computed distribution of participants’ CVAI values for the years 2011 and 2015 is illustrated in Figure S1. As CVAI stands as a relatively novel indicator, there are currently no universally recognized standards to categorize its levels. Therefore, we employed receiver operating characteristic curves to convert it into a binary variable using the Youden index. Cutoff values of 88.42 for females and 101.80 for males were identified, defining high and low CVAI groups within the baseline data. Acknowledging the variability of CVAI over time, for a more comprehensive investigation into the relationship between CVAI changes and outcomes, we utilized data from two biochemical variables in 2011 and 2015. Using specific cutoff values, participants were grouped into the following four categories: (1) low-low group, representing individuals maintaining a low CVAI status over the course of the study; (2) low-high group, characterized by a baseline low CVAI that transitioned to high CVAI over the course of the study; (3) high-low group, displaying a high baseline CVAI that decreased to low CVAI throughout follow-up; and (4) high-high group, demonstrating a sustained high CVAI status throughout follow-up.

### Outcome and follow-up

Our primary outcome consisted of incident cardiovascular and cerebrovascular diseases, defined as various cardiovascular illnesses, stroke, or any combination of them. Cardiovascular diseases include myocardial infarction, coronary artery atherosclerosis, angina pectoris, congestive heart failure, and other cardiac disease [42, 43]. Stroke comprised ischemic and hemorrhagic cerebrovascular diseases. Primary outcomes were diagnosed by qualified healthcare professionals based on medical evaluations and confirmed by self-reported patient declarations. Participants were followed from their enrollment in 2011 until the first occurrence of a cardiovascular or cerebrovascular event, death, or the conclusion of the research duration, whichever event occurred first.

### Covariables

Our study considered various covariables based on prior research and clinical expertise. These included population characteristics (age, gender, residency, marriage status, and educational background), socioeconomic status (per capita expenditures), health behaviors (smoking, alcohol consumption), concomitant comorbidities (hypertension, diabetes mellitus, lung disease, liver disease, digestive disease), and biochemical variables (TC, LDL, HbA1c, Scr, BUN). Among these confounders, age and biochemical variables were treated as continuous variables, while others were categorical variables.

Demographic variables were collected using structured questionnaires aided by CAPI, ensuring data reliability through rigorous quality control measures such as audio review, follow-up calls, and iterative data verification. Socioeconomic status was evaluated using the natural logarithm transformation of per capita expenditures (PCE), measured in Chinese Yuan (CNY). We categorized ln(PCE) into three levels based on tertiles to represent low, moderate, and high economic statuses across all participants [44]. Health behaviors such as smoking and alcohol usage were assessed based on individuals’ current behavior status. According to the diagnostic criteria established by the American Diabetes Association (ADA) in 2014 [45], the definition of diabetes mellitus is as follows: fasting blood glucose level ≥ 126 mg/dL (7 mmol/L), and/or random blood glucose level ≥ 200 mg/dL (11.1mmol/L), and/or HbA1c level ≥ 6.5%, and/or self-reported diagnosis by the patient, and/or use of hypoglycemic medications. Hypoglycemic medications include insulin and other oral hypoglycemic agents. Hypertension was defined by self-reported diagnosis or the use of antihypertensive medications. Medication usage, including both traditional Chinese and Western medicines, was queried immediately after patients were asked whether they had the respective diseases. Furthermore, biochemical variables were obtained according to previously detailed methods.

### Statistical analyses

Data preprocessing, subsequent analyses, and result visualization were conducted using R (version 4.2.3). Statistical significance was determined for *p*-values less than 0.05 with a two-tailed assessment. The characteristics of the study participants were grouped based on the baseline patterns and transition patterns of CVAI. Continuous variables were presented as mean ± standard deviation and were analyzed using ANOVA, while numbers (percentages) represented categorical variables and were evaluated through chi-square tests.

Firstly, Kaplan-Meier curves were used to visualize cumulative incidence proportions over time for each exposure group, and statistical assessment of group differences was conducted utilizing log-rank tests. Cox proportional hazard models were employed to evaluate the hazard ratios (HRs) and 95% confidence intervals for cardiovascular and cerebrovascular disease risk across different CVAI groups. To ensure the robustness of the results, four Cox models were established, integrating baseline patterns and transition patterns of CVAI. Model 1 represented the unadjusted original model without correcting for any covariables. Model 2 adjusts for age, gender (male/female), residence (urban/rural), marital status (married and cohabiting/single), educational level (middle school or below/high school or above), the ln(PCE) (bottom tertile/middle tertile/top tertile). Model 3, further adjusting for smoking (yes/no), alcohol consumption (yes/no), hypertension (yes/no), diabetes (yes/no), lung disease (yes/no), liver disease (yes/no), digestive disease (yes/no) on top of Model 2. Model 4 encompassed adjustments for all confounding factors in Model 3 and additionally controlled for TC, LDL, HbA1c, Scr, and BUN. Schoenfeld residual tests were applied to verify the proportional hazard assumption across all models (Table S2). Additionally, adjusted variables underwent multicollinearity assessment using variance inflation factors (VIF), revealing no substantial evidence of multicollinearity (Figure S2). The missing value distribution for each variable is presented in Figure S3 and Table S3. Under the missing at random assumption, multiple imputation (random forest) was employed to address missing data, accomplished through the ‘mice’ package.

Restricted cubic spline (RCS) curves were employed to explore the dose-response relationship between CVAI and primary outcomes, and the knots were set at the 5th, 35th, 65th, and 95th percentiles, respectively. Furthermore, subgroup analyses were performed according to age, gender, BMI, residence, marital status, education, smoking status, alcohol consumption status, hypertension, and diabetes. Considering the disparity in disease risk experienced by individuals of different socioeconomic statuses across various age groups, we divided participants into six groups based on age and ln(PCE) for analyses. To ensure robustness in the results, several sensitivity analyses were performed. Primarily, a reanalysis was conducted using logistic regression models, utilizing odds ratios (ORs) to depict the strength of the association between CVAI and cardiovascular and cerebrovascular diseases. Secondly, to further avoid the confusion of reverse causation, we additionally excluded individuals who experienced the primary outcome events during the second follow-up. Furthermore, we analyzed the population without concomitant comorbidities to mitigate the impact of reverse causation. Thirdly, individuals diagnosed with cancer at baseline were also excluded. Finally, E-values were computed to evaluate the possible effects of unmeasured confounders on the causal conclusions of the study. A higher E-value indicates a stronger association of the unmeasured confounder needed to explain the observed effect [46].

## Results

### Study population

In the cohort of 17,708 individuals, we initially excluded 10,531 participants based on demographics and health information from the wave 2011. Following the criteria for inclusion and exclusion, 7717 individuals and 4993 individuals were respectively enrolled based on baseline patterns and transition patterns with a 9-year follow-up period. The regional distribution of study participants is illustrated in Figure S4. Details of the baseline characteristics of excluded and included participants can be found in Table S4. The population demographics categorized by baseline patterns and transition patterns are presented in Table 1. The demographic data after multiple imputation are displayed in Table S5. Compared to the low CVAI group, the high CVAI group had a higher proportion of elderly females (62.5%), lower rates of smoking (22.6%), alcohol consumption (28.2%),urban residents (40.2%), higher socioeconomic status (36.3%), and higher educational attainment (9.7%). Regarding concomitant comorbidities, they displayed a greater incidence of hypertension and diabetes, yet lower incidence rates of pulmonary and digestive disorders. In the transition patterns of CVAI, the high-high subgroup showed consistent characteristics with the baseline, including a higher proportion of elderly females residing in urban areas and individuals with higher socioeconomic status. However, no significant differences were observed concerning marital status and educational levels. The low-low subgroup exhibited a higher percentage of individuals who smoked and consumed alcohol, while the high-low subgroup showed larger proportions in diabetes prevalence and mean creatinine levels. In contrast, the high-high subgroup exhibited a higher prevalence of hypertension and elevated levels of BMI, waist circumference, LDL, TC, TG, and HbA1c compared to other subgroups. Additional features regarding gender classification and outcome categorization within the baseline population are detailed in the appendix. (Table S6-S7)

**Table 1:** Characteristics of participants stratified by baseline patterns and transition patterns of CVAI

	Baseline patterns				Transition patterns					
	Overall	Low	High	*P* Value	Overall	Low-Low	Low-High	High-Low	High-High	*P* Value
n	7,717	3,995	3,722		4,993	1,859	696	183	2,255	
Baseline CVAI	94.04 ± 38.87	64.09 ± 21.15	126.18 ± 25.62	<0.001	94.71 ± 38.50	59.89 ± 21.32	77.85 ± 13.70	111.38 ± 18.56	127.26 ± 25.53	<0.001
Age, years	58.82 ± 9.31	57.49 ± 8.87	60.24 ± 9.56	<0.001	58.33 ± 8.65	57.45 ± 8.45	56.51 ± 8.21	58.52 ± 9.15	59.61 ± 8.73	<0.001
Gender, n (%)				<0.001						<0.001
Male	3671 (47.6)	2276 (57.0)	1395 (37.5)		2335 (46.8)	1172 (63.0)	270 (38.8)	108 (59.0)	785 (34.8)	
Female	4046 (52.4)	1719 (43.0)	2327 (62.5)		2658 (53.2)	687 (37.0)	426 (61.2)	75 (41.0)	1470 (65.2)	
Residence, n (%)				<0.001						<0.001
Urban	2593 (33.6)	1097 (27.5)	1496 (40.2)		1615 (32.3)	491 (26.4)	191 (27.4)	57 (31.1)	876 (38.8)	
Rural	5124 (66.4)	2898 (72.5)	2226 (59.8)		3378 (67.7)	1368 (73.6)	505 (72.6)	126 (68.9)	1379 (61.2)	
Marital, n (%)				0.044						0.871
Married and cohabiting	6463 (83.8)	3379 (84.6)	3084 (82.9)		4289 (85.9)	1602 (86.2)	598 (85.9)	160 (87.4)	1929 (85.5)	
Single	1254 (16.2)	616 (15.4)	638 (17.1)		704 (14.1)	257 (13.8)	98 (14.1)	23 (12.6)	326 (14.5)	
Ln(PCE)^a b^, n (%)				<0.001						0.069
Bottom tertile	2221 (33.4)	1240 (36.0)	981 (30.5)		1516 (35.1)	609 (37.9)	207 (34.1)	51 (32.1)	649 (33.3)	
Middle tertile	2219 (33.3)	1153 (33.5)	1066 (33.1)		1444 (33.4)	528 (32.9)	207 (34.1)	59 (37.1)	650 (33.3)	
Top tertile	2218 (33.3)	1049 (30.5)	1169 (36.3)		1363 (31.5)	470 (29.2)	193 (31.8)	49 (30.8)	651 (33.4)	
Education, n (%)				0.097						0.546
Middle School or below	7013 (90.9)	3652 (91.4)	3361 (90.3)		4571 (91.5)	1713 (92.1)	633 (90.9)	164 (89.6)	2061 (91.4)	
High school or above	704 (9.1)	343 (8.6)	361 (9.7)		422 (8.5)	146 (7.9)	63 (9.1)	19 (10.4)	194 (8.6)	
Alcohol drinking, n (%)				<0.001						<0.001
Non-drinker	5092 (66.0)	2418 (60.5)	2674 (71.8)		3319 (66.5)	1084 (58.3)	484 (69.5)	111 (60.7)	1640 (72.7)	
Drinker	2625 (34.0)	1577 (39.5)	1048 (28.2)		1674 (33.5)	775 (41.7)	212 (30.5)	72 (39.3)	615 (27.3)	
Smoking^a^, n (%)				<0.001						<0.001
Non-smoker	5271 (68.5)	2399 (60.2)	2872 (77.4)		3444 (69.2)	1045 (56.2)	504 (72.9)	117 (63.9)	1778 (79.1)	
smoker	2424 (31.5)	1585 (39.8)	839 (22.6)		1536 (30.8)	813 (43.8)	187 (27.1)	66 (36.1)	470 (20.9)	
Hypertension^a^, n (%)	1812 (23.6)	557 (14.0)	1255 (33.8)	<0.001	1192 (24.0)	225 (12.2)	133 (19.2)	36 (19.8)	798 (35.5)	<0.001
Diabetes mellitus^a^, n (%)	1240 (16.3)	418 (10.6)	822 (22.4)	<0.001	790 (16.0)	193 (10.5)	61 (8.9)	48 (26.5)	488 (21.9)	<0.001
Lung disease^a^, n (%)	835 (10.8)	471 (11.8)	364 (9.8)	0.005	507 (10.2)	212 (11.4)	72 (10.3)	19 (10.4)	204 (9.1)	0.096
Liver disease^a^, n (%)	261 (3.4)	151 (3.8)	110 (3.0)	0.053	156 (3.1)	62 (3.3)	29 (4.2)	8 (4.4)	57 (2.5)	0.097
Digestive disease^a^, n (%)	1903 (24.7)	1086 (27.3)	817 (22.0)	<0.001	1178 (23.6)	493 (26.6)	166 (23.9)	44 (24.0)	475 (21.1)	0.001
BMI, kg/m2	23.26 ± 3.53	21.13 ± 2.37	25.56 ± 3.11	<0.001	23.41 ± 3.50	20.74 ± 2.15	22.59 ± 2.15	23.68 ± 2.49	25.85 ± 3.04	<0.001
WC, cm	84.81 ± 9.86	78.04 ± 6.20	92.07 ± 7.66	<0.001	85.13 ± 9.76	77.18 ± 5.90	81.30 ± 5.76	88.52 ± 5.50	92.58 ± 7.50	<0.001
TC^a^, mg/dL	193.61 ± 38.59	188.22 ± 36.49	199.39 ± 39.93	<0.001	193.54 ± 38.38	186.45 ± 35.50	192.18 ± 35.72	192.92 ± 39.66	199.86 ± 40.27	<0.001
TG, mg/dL	131.24 ± 108.05	97.04 ± 60.79	167.95 ± 132.81	<0.001	133.20 ± 108.50	94.78 ± 54.39	102.18 ± 54.09	152.93 ± 84.49	172.85 ± 137.98	<0.001
HDL, mg/dL	51.57 ± 15.36	57.41 ± 15.44	45.30 ± 12.55	<0.001	51.21 ± 15.32	57.94 ± 16.04	54.86 ± 13.50	46.48 ± 13.15	44.91 ± 12.43	<0.001
LDL^a^, mg/dL	116.43 ± 34.88	113.04 ± 32.13	120.08 ± 37.27	<0.001	116.21 ± 34.68	110.99 ± 31.50	118.80 ± 31.03	115.65 ± 36.51	119.76 ± 37.49	<0.001
HbA1c^a^, %	5.26 ± 0.80	5.15 ± 0.68	5.37 ± 0.90	<0.001	5.25 ± 0.80	5.14 ± 0.69	5.15 ± 0.59	5.30 ± 0.93	5.37 ± 0.90	<0.001
Scr^a^, mg/dL	0.78 ± 0.24	0.78 ± 0.20	0.78 ± 0.27	0.858	0.77 ± 0.18	0.78 ± 0.17	0.75 ± 0.19	0.80 ± 0.20	0.76 ± 0.18	<0.001
BUN^a^, mg/dL	15.76 ± 4.58	15.95 ± 4.75	15.56 ± 4.38	<0.001	15.64 ± 4.33	16.00 ± 4.45	15.55 ± 4.48	15.13 ± 4.18	15.42 ± 4.17	<0.001

Mean ± SD for continuous variables and counts (percentages) for categorical variables

CVAI Chinese visceral adiposity index, PCE per capita expenditures, BMI body mass index, WC waist circumference, TC total cholesterol, TG triglycerides, HDL high-density lipoprotein, LDL low-density lipoprotein, HbA1c glycosylated hemoglobin A1c, Scr serum creatinine, BUN blood urea nitrogen

High group: CVAI ≥ 88.42 (females), ≥ 101.80 (males); Low group: CVAI < 88.42 (females), < 101.80 (males)

^a^ Data for some participants were missing

^b^ The unit of per capita expenditures is Chinese Yuan (CNY).

### Baseline patterns and primary outcomes

**Fig. 2:**
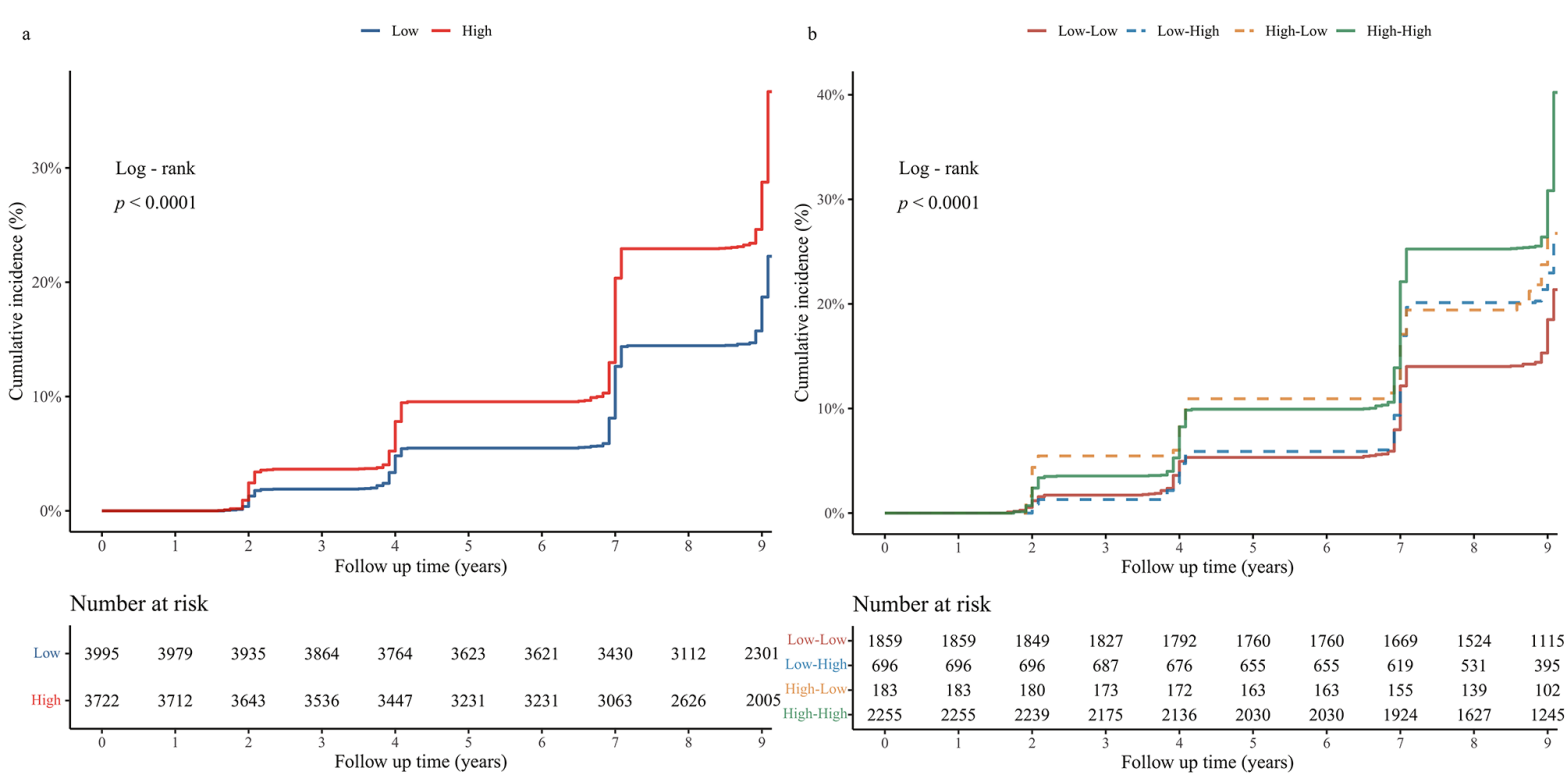
Kaplan–Meier curves for the cumulative incidence of cardiovascular and cerebrovascular diseases. (**a**) Kaplan–Meier curves for the cumulative incidence of cardiovascular and cerebrovascular diseases in different baseline patterns. (**b**) Kaplan–Meier curves for the cumulative incidence of cardiovascular and cerebrovascular diseases in different transition patterns. High group: CVAI ≥ 88.42 (females), ≥ 101.80 (males); Low group: CVAI < 88.42 (females), < 101.80 (males)

**Fig. 3:**
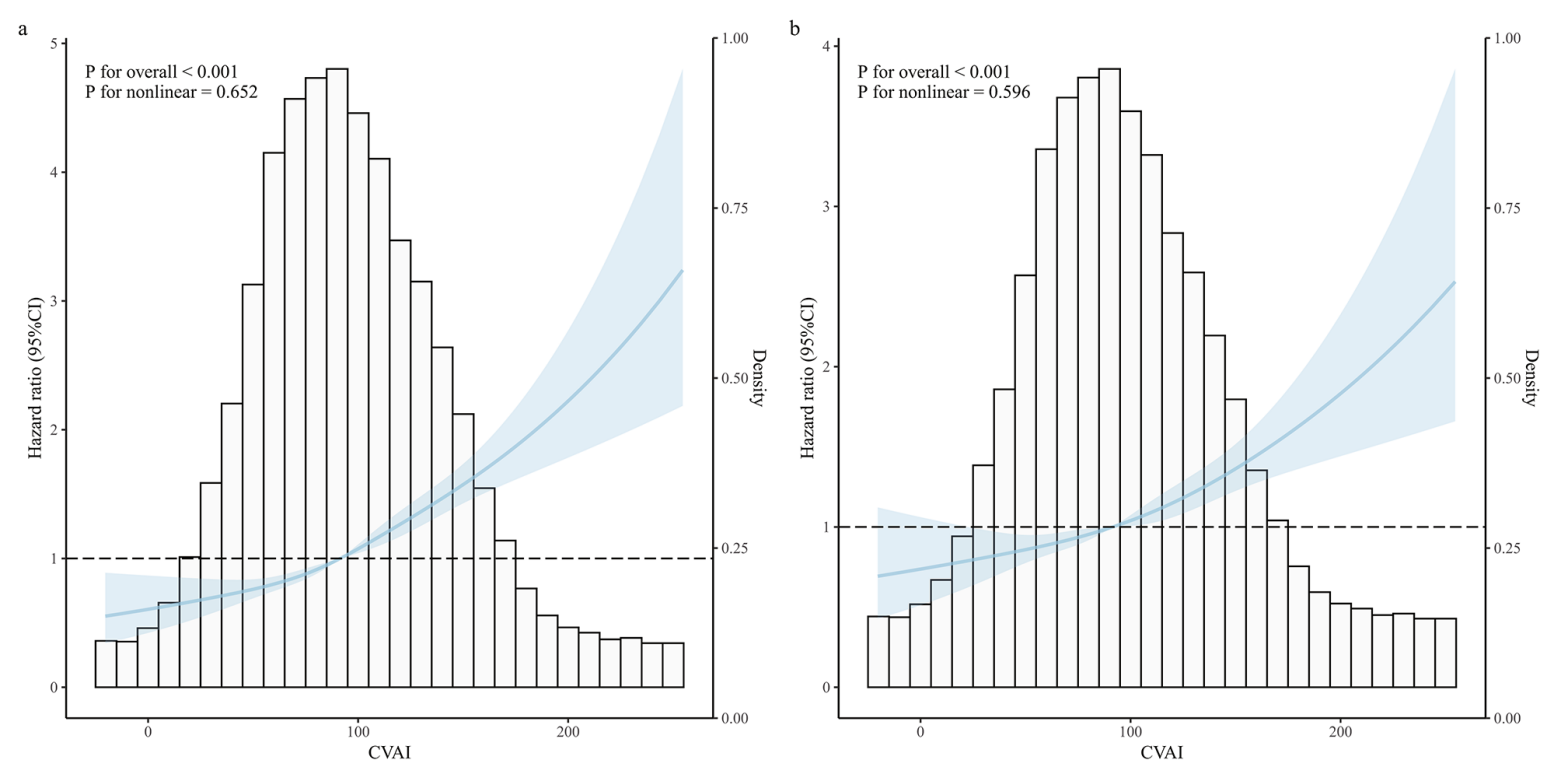
Restricted cubic spline curves for cardiovascular and cerebrovascular diseases before/after covariables adjustment. (**a**) Restricted cubic spline curves for cardiovascular and cerebrovascular diseases by CVAI before covariables adjustment. (**b**) Restricted cubic spline curves for cardiovascular and cerebrovascular diseases by CVAI after adjusted for age, gender, residence, marital status, educational level, ln(PCE), smoking, alcohol consumption, hypertension, diabetes, lung disease, liver disease, digestive disease, TC, LDL, HbA1c, Scr, BUN. CVAI Chinese visceral adiposity index, PCE per capita expenditures, TC total cholesterol, LDL low-density lipoprotein, HbA1c glycosylated hemoglobin A1c, Scr serum creatinine, BUN blood urea nitrogen. High group: CVAI ≥ 88.42 (females), ≥ 101.80 (males); Low group: CVAI < 88.42 (females), < 101.80 (males)

In the baseline patterns analysis, a total of 1761 individuals (22.82%) experienced outcomes, with 711 (17.80%) in the low CVAI group and 1050 (28.21%) in the high CVAI group. Figure 2a illustrates the cumulative incidence rates of primary outcomes among different groups categorized by CVAI baseline patterns throughout the observation period. In contrast to the low CVAI group, we observed a markedly higher cumulative incidence rate of primary outcomes in the high CVAI group, with statistically significant differences perceived between the groups (log-rank test *p* < 0.001). As depicted in Table 2, the high CVAI group exhibited a HR of 1.68 (95%CI: 1.52–1.84) for the occurrence of primary outcomes in model 1. It exhibited an elevated risk even after adjusting for confounding factors. In partially adjusted models 2 and 3, the HR values were 1.54 (95%CI: 1.39–1.70) and 1.41 (95%CI: 1.27–1.57), respectively, and in the fully adjusted model 4, the HR values remained significant [HR = 1.38 (95%CI: 1.25–1.54)]. When CVAI was treated as a continuous variable, similar outcomes were observed with each one-standard-deviation increase in CVAI. Furthermore, both unadjusted and fully adjusted RCS curves unveiled a significant dose-response relationship between CVAI and the occurrence of primary outcomes (Fig. 3), indicating a notable positive association between elevated CVAI levels and the risk of outcomes.

**Table 2:** The association of CVAI with cardiovascular and cerebrovascular diseases

CVAI^a^	Total N	No. of events (Incident rate^b^)	Model 1		Model 2		Model 3		Model 4	
			HR (95% CI)	*P* value	HR (95% CI)	*P* value	HR (95% CI)	*P* value	HR (95% CI)	*P* value
Continues										
Per SD increase	7717	1761 (22.82)	1.32 (1.27-1.39)	<0.001	1.28 (1.22-1.34)	<0.001	1.22 (1.15-1.28)	<0.001	1.20 (1.14-1.27)	<0.001
Baseline patterns										
Low	3995	711 (17.80)	Ref.		Ref.		Ref.		Ref.	
High	3722	1050 (28.21)	1.68 (1.52-1.84)	<0.001	1.54 (1.39-1.70)	<0.001	1.41 (1.27-1.57)	<0.001	1.38 (1.25-1.54)	<0.001
Transition patterns										
Low - Low	1859	335 (18.02)	Ref.		Ref.		Ref.		Ref.	
Low - High	696	161 (23.13)	1.31 (1.09-1.58)	0.005	1.28 (1.06-1.55)	0.010	1.23 (1.01-1.49)	0.035	1.22 (1.01-1.47)	0.044
High - Low	183	46 (25.14)	1.47 (1.08-2.00)	0.015	1.42 (1.04-1.93)	0.027	1.36 (1.00-1.86)	0.050	1.34 (0.98-1.83)	0.065
High - High	2255	719 (31.88)	1.90 (1.67-2.16)	<0.001	1.74 (1.52-1.99)	<0.001	1.55 (1.35-1.79)	<0.001	1.51 (1.31-1.74)	<0.001

Model 1: unadjusted

Model 2: adjusted for age, gender, residence, marital status, educational level, ln(PCE)

Model 3: model 2 + further adjusted for smoking, alcohol consumption, hypertension, diabetes, lung disease, liver disease, digestive disease

Model 4: model 3 + further adjusted for TC, LDL, HbA1c, Scr, BUN

CI confidence interval, CVAI Chinese visceral adiposity index, PCE per capita expenditures, TC total cholesterol, LDL low-density lipoprotein, HbA1c glycosylated hemoglobin A1c, Scr serum creatinine, BUN blood urea nitrogen

^a^ High group: CVAI ≥ 88.42 (females), ≥ 101.80 (males); Low group: CVAI < 88.42 (females), < 101.80 (males)

^b^ Incident rate was presented as a percentage

**Fig. 4:**
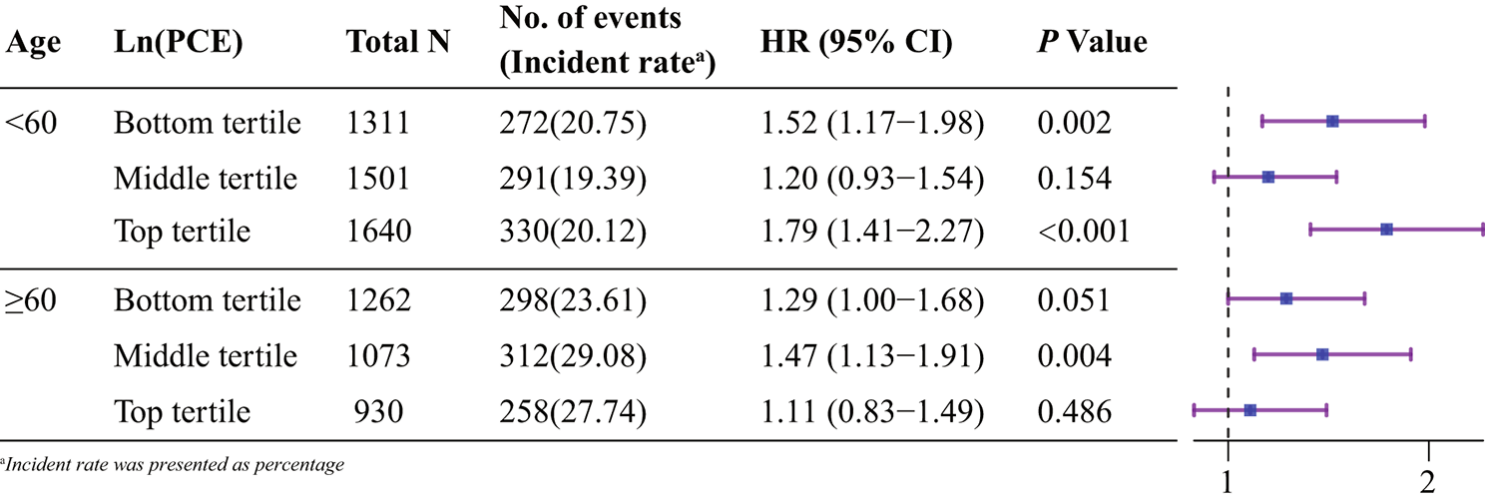
Multivariable-adjusted HRs (95% CIs) for cardiovascular and cerebrovascular diseases according to different age groups and different ln(PCE) tertiles. The models were adjusted for gender, residence, marital status, educational level, smoking, alcohol consumption, hypertension, diabetes, lung disease, liver disease, digestive disease, TC, LDL, HbA1c, Scr, BUN. CI confidence interval, CVAI Chinese visceral adiposity index, PCE per capita expenditures, TC total cholesterol, LDL low-density lipoprotein, HbA1c glycosylated hemoglobin A1c, Scr serum creatinine, BUN blood urea nitrogen

Additionally, we further explore the relationship between CVAI and primary outcomes across various age groups and different ln(PCE) tertiles (Fig. 4). In middle-aged individuals, we observed divergent disease risks between those with low socioeconomic status [HR = 1.52 (95%CI: 1.17–1.98)] and high socioeconomic status [HR = 1.79 (95%CI: 1.41–2.27)], while the hazard ratios for individuals in the intermediate socioeconomic status group losing statistical significance [HR = 1.20 (95%CI: 0.93–1.54)]. However, among older adults, individuals with moderate socioeconomic status exhibited high disease risks [HR = 1.47 (95%CI: 1.13–1.91)], while the hazard ratios for those at the extremes of socioeconomic status lacked statistical significance [HR = 1.29 (95%CI: 1.00-1.68) and HR = 1.11 (95%CI: 0.83–1.49), respectively].

### Transition patterns and primary outcomes

Turning to the transition patterns analysis, outcomes were observed in 1261 individuals (25.26%), distributed as follows: 335 (18.02%) in the low-low group, 161 (23.13%) in the low-high group, 46 (25.14%) in the high-low group, and 719 (31.88%) in the high-high group. The Kaplan-Meier curve in Fig. 2b revealed interesting findings. Broadly, the cumulative incidence rates of cardiovascular and cerebrovascular diseases were notably higher in the high-initiated groups compared to the low-initiated groups. Specifically, the high-initiated group consistently surpassed the low-initiated groups, while the high-low group consistently remained higher than the low-low group. Notably, the second CVAI measurement occurred in the 48th month after enrollment. When segmenting the data at these time points, a distinctive trend became apparent: starting from the 48th month, the rate of cardiovascular and cerebrovascular disease incidence in the high-low group exhibited a gradual slowdown, while in the high-initiated group, the rate accelerated. As a result, the gap between the high-high and high-low groups widened progressively, leading to a significantly lower incidence rate in the high-low group compared to the high-high group. Simultaneously, the low-high group experienced an accelerated growth in incidence rate from the 48th month onwards, whereas the incidence rate in the low-initiated group remained relatively stable. Post-48 months, the disparity between the low-initiated groups gradually widened, resulting in a notably higher incidence rate in the low-high group compared to the low-low group.

In terms of the association between CVAI transition patterns and the risk of primary outcomes, all three groups displayed varying degrees of increased risk for outcomes compared to the low-low group in model 1. Specifically, the high-high group exhibited the highest risk (HR = 1.90, 95% CI: 1.67–2.16), followed by the high-low group (HR = 1.47, 95% CI: 1.08-2.00), with the low-high group showing a comparatively lower risk (HR = 1.31, 95% CI: 1.09–1.58). The same sequence applies in several other models. In model 2, although the HR values for each group decreased, they still indicated higher risk compared to the low-low group and remained statistically significant. However, in models 3 and 4, although the point estimate for the high-low group indicated increased risk, the confidence intervals did not reach statistical significance. Conversely, the high-high group maintained statistically significant increased risk (model 4: HR = 1.51, 95% CI 1.31–1.74), and similarly, the low-high group also demonstrated statistically significant higher risk (model 4: HR = 1.22, 95% CI 1.01–1.47).

### Subgroup analyses

**Fig. 5:**
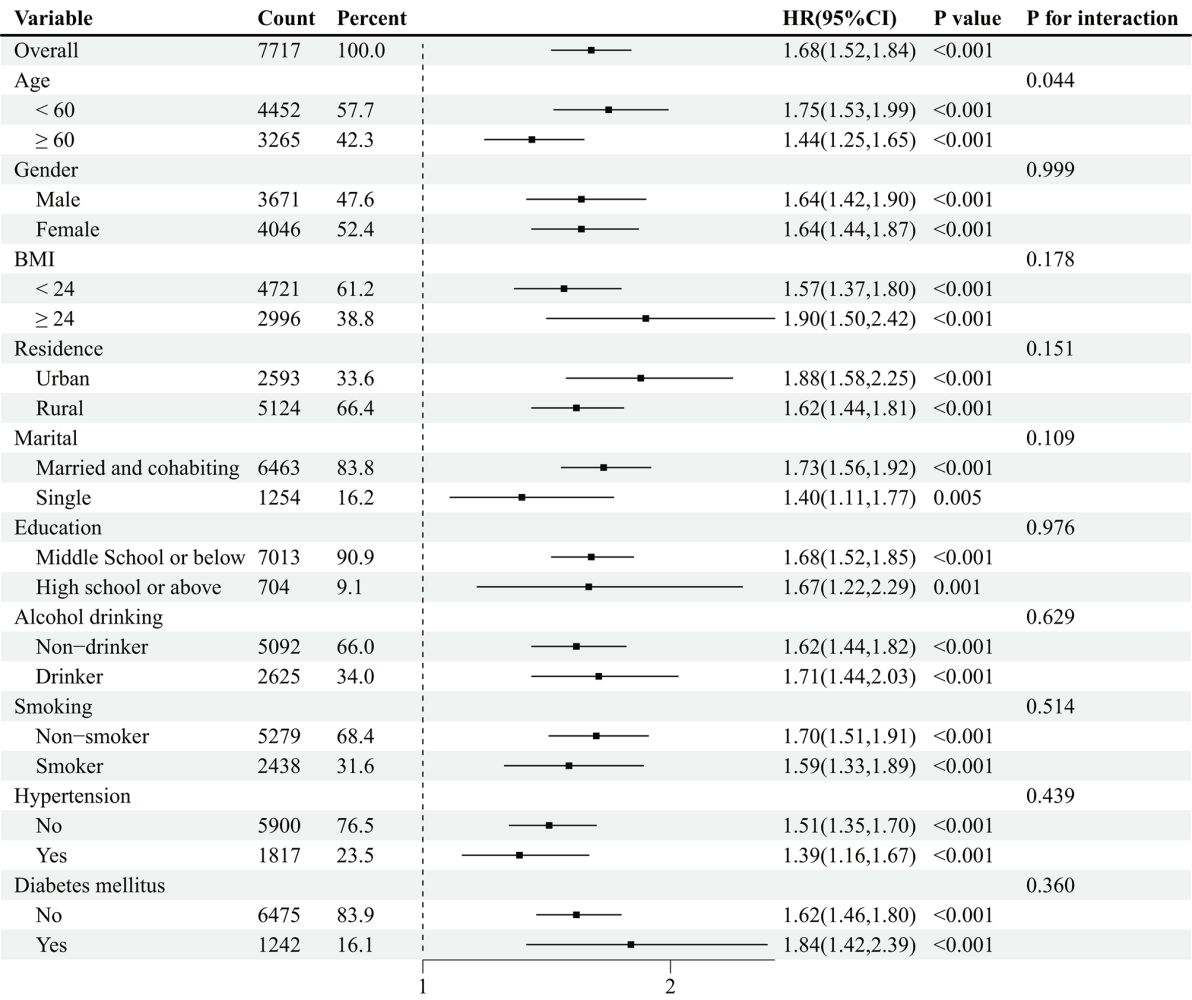
Subgroup and interaction analyses between the CVAI and cardiovascular and cerebrovascular diseases. High group: CVAI ≥ 88.42 (females), ≥ 101.80 (males); Low group: CVAI < 88.42 (females), < 101.80 (males)

The subgroup analyses depicted in Fig. 5 further illustrate the hazard ratios and interactions of high CVAI on the incidence rates of cardiovascular and cerebrovascular diseases across ten predefined subgroups. Consistency was observed across several predefined subgroups (all interaction p-values > 0.05), except for age, where interaction was slightly noted (p for interaction = 0.044). Despite the relatively weak interaction observed, subgroup analyses revealed that high CVAI increased the risk of cardiovascular and cerebrovascular diseases in both age subgroups. Interestingly, the hazard ratio for developing these diseases due to high CVAI was higher in the middle-aged population compared to the elderly, despite the subtlety of this interaction.

### Sensitivity analyses

Our results exhibit robustness across a range of sensitivity analyses. Primarily, logistic regression revealed that the high CVAI group confronted a 1.52-fold increased risk compared to the low CVAI group. Compared to the low-low transition pattern, the high-high transition pattern indicated a 67% increase in the risk of outcomes, while the low-high transition pattern showed a 1.26-fold increase; all these results were statistically significant (Table S8). Secondly, when individuals who experienced cardio-cerebrovascular diseases either at baseline or during the second longitudinal monitoring were excluded, our primary findings remained consistent (high CVAI: HR = 1.37, 95% CI 1.22–1.54; high-high CVAI: HR = 1.56, 95% CI 1.33–1.84; low-high CVAI: HR = 1.34, 95% CI 1.09–1.65) (Table S9). Furthermore, our analysis of the population without concomitant comorbidities shows findings consistent with the primary analysis results (Table S10), with hazard ratios even higher than those in the primary results (high CVAI: HR = 1.27, 95% CI 1.15–1.40; high-high CVAI: HR = 1.88, 95% CI 1.47–2.39; low-high CVAI: HR = 1.51, 95% CI 1.11–2.07). Thirdly, following the exclusion of individuals diagnosed with cancer at baseline, the results remained robust (high CVAI: HR = 1.38, 95% CI 1.24–1.54; high-high CVAI: 1.52, 95% CI 1.31–1.75; low-high CVAI: HR = 1.23, 95% CI 1.02–1.49) (Table S11). Finally, as depicted in Figure S5, the analysis of e-values signifies substantial unmeasured confounding that could account for our research findings. This further underscores the robustness of our results.

## Discussion

In this nationwide, large-scale cohort study spanning approximately 9 years of observation, a higher CVAI remained significantly associated with an elevated risk of incident cardiovascular and cerebrovascular diseases after adjusting for confounding factors. Middle-aged individuals with both low and high socioeconomic statuses, as well as adults with moderate socioeconomic status, exhibit varying degrees of disease risk associated with high CVAI levels. Moreover, RCS curves demonstrated a direct association between higher CVAI levels and an increased risk of cardiovascular and cerebrovascular diseases. Kaplan-Meier curves highlighted notably higher cumulative incidence rates among individuals in the high CVAI group compared to their counterparts in the low CVAI group. Regarding transition patterns, the low-low group showed the lowest cumulative incidence rates, while the high-high group exhibited the highest rates. Transitioning from low to high CVAI led to a substantial increase in cumulative incidence rates compared to maintaining a low CVAI level. Conversely, transitioning from high to low CVAI corresponded to a decrease in cumulative incidence rates compared to maintaining a high CVAI level. In assessing the association between CVAI transition patterns and the risk of cardio-cerebrovascular diseases, maintaining consistently high CVAI levels showed a risk increase of over 51% compared to consistently maintaining low CVAI levels. Transitioning from low to high CVAI was associated with a 22% higher risk of cardiovascular and cerebrovascular diseases, whereas transitioning from high to low CVAI did not maintain statistical significance in fully adjusted models. This emphasizes the importance of initially maintaining lower CVAI levels and monitoring to sustain these levels, potentially aiding primary prevention of cardiovascular and cerebrovascular diseases. Further interventional studies may be necessary to ascertain whether improving CVAI can reduce the risk of these diseases.

According to our information, this is the first nationwide study in China exploring the association between CVAI and the emergence of cardiovascular and cerebrovascular diseases among middle-aged and elderly populations. Previous studies, limited to specific regions in China, typically focused on particular patient groups, predominantly covering myocardial infarction and stroke, without considering the full spectrum of cardiovascular diseases. Nevertheless, their main findings were consistent. A study by Qiao et al. [41], using data from Xinjiang, China, demonstrated the association of various abdominal fat indicators with myocardial infarction and stroke, highlighting CVAI as a potentially valuable marker for identifying high cardiovascular risk in patients with type 2 diabetes. Interestingly, Wang et al. [47] found in the southwestern Chinese population that CVAI serves as a robust predictive indicator for cardiovascular diseases in females, with significant predictive power observed only among female participants, while a gender difference was not evident in our study. Wu et al. [48] concluded from their study on the hypertensive population that prolonged presence in high CVAI and greater duration of exposure to high CVAI could elevate the risk of cardiovascular diseases. Notably, early accumulation of CVAI posed a higher risk compared to later accumulation, which indirectly supported our study findings.

While there are various anthropometric measures reflecting obesity, BMI retains its primary status in estimating overall obesity, invariably associated with heightened probabilities of cardiovascular and cerebrovascular diseases [49, 50]. However, BMI lacks information about regional body fat distribution. Studies indicate that excessive fat deposition has metabolic consequences, with visceral obesity signaling dysfunctional adipose tissue and ectopic fat infiltration. Central adiposity poses a greater risk for cardiovascular diseases and type 2 diabetes compared to peripheral fat accumulation [51, 52]. Lifestyle interventions inducing weight loss often prioritize mobilizing visceral fat. Research highlights the independent role of localized fat content in mortality and cardiovascular diseases [53]. Our findings support this notion, demonstrating that BMI, waist circumference, LAP, and similar measures don’t fully capture the interrelation between obesity and susceptibility to cardiovascular and cerebrovascular diseases. We compared various traditional measures of obesity and found that CVAI exhibits superior predictive capability for cardiovascular and cerebrovascular diseases compared with VAI, LAP, WC, and BMI within the middle-aged and elderly population in China (Figure S6, Table S12).

Dysfunction in adipose tissue affects its generation, differentiation, and expansion, ultimately affecting lipid storage and retention. VAT deposition has been consistently associated with lipid accumulation in normal lean tissues, including the heart, liver, pancreas, and skeletal muscles [15]. The precise mechanisms linking visceral fat deposition to cardiovascular and cerebrovascular diseases remain unclear. There are several possible explanations for their association. Firstly, as an active endocrine organ, VAT releases a plethora of inflammatory cytokines such as IL-6 and TNF-α, thereby inducing a low-grade inflammatory state and insulin resistance. Elevated levels of insulin resistance increase the risk of developing cardiovascular diseases, ultimately leading to cardiac metabolic disorders [54, 55]. Secondly, adipocytes and matrix cells within VAT generate various bioactive substances termed adipokines [56, 57], where the decrease of anti-inflammatory adiponectin, such as adiponectin, and the increase of pro-inflammatory factors, such as resistin and leptin, contribute to endothelial damage, heightened vascular permeability, exacerbating inflammatory responses, lipid accumulation, and several other alterations that exacerbate adverse cardiovascular events [58–60]. These changes potentially impair vascular dilation, aggravate atherosclerosis, and initiate myocardial fibrosis and myocardial infarction [61, 62].

This study provides compelling evidence that CVAI serves as an obesity index strongly associated with cardiovascular and cerebrovascular diseases, which continue to be one of the leading causes of morbidity and mortality in both developed and developing nations [63, 64]. Visceral fat accumulation is a key indicator of adipose tissue dysfunction and is strongly correlated with cardiovascular and cerebrovascular disease risk compared to overall obesity [15]. CVAI, derived from readily available clinical data through simple calculations, offers a feasible means to enhance existing risk assessments and enables precise identification of individuals at high risk for cardiovascular and cerebrovascular diseases. Consequently, considering interventions aimed at reducing abdominal fat deposition may have profound implications for the care, outcomes, and utilization of healthcare resources for high-risk patients. Weight loss through healthy lifestyle changes typically induces preferential mobilization of visceral fat [65]. Therefore, encouraging lifestyle modifications such as increased physical activity and dietary changes to promote a healthier distribution of fat could represent a crucial strategy for improving population health.

The strength of this study includes its large and representative sample size coupled with an extensive follow-up duration, enabling a thorough longitudinal investigation between these factors. Adjusting for multiple covariables helps minimize the impact of confounding factors. Moreover, our findings underwent comprehensive sensitivity analyses to ensure their reliability and robustness. However, our study also harbors limitations. Primarily, certain variables in our research, such as smoking and alcohol consumption, relied on self-reporting or survey questionnaires, potentially susceptible to recall bias. Secondly, given the observational nature of our study, establishing causal relationships may necessitate further interventional experiments. Thirdly, despite adjusting for various confounding factors, we cannot entirely eliminate unmeasurable or unknown confounders, potentially impacting the associations observed in this study. Nonetheless, population homogeneity and comprehensive data regarding risk factors have substantially minimized potential confounding. The E-value suggests that substantial unmeasured confounding would be necessary to explain the associations observed in this research. Finally, as the CVAI is tailored specifically for the Chinese population, our study predominantly focuses on middle-aged and elderly individuals in China. We earnestly hope that future research will surpass the limitations of this study and unveil more persuasive findings in a broader population.

## Conclusions

In this nine-year longitudinal population-based study, high CVAI demonstrated clinically significant associations with cardiovascular and cerebrovascular diseases compared to low CVAI. Transitioning from low to high CVAI and maintaining high CVAI significantly increased the risk of developing cardiovascular and cerebrovascular diseases. Our research further substantiates the association between visceral fat and cardiovascular and cerebrovascular diseases, while also supporting CVAI as a valuable indicator for these conditions in middle-aged and elderly populations.

## Electronic supplementary material

Below is the link to the electronic supplementary material.

Supplementary Material 1

## Data Availability

No datasets were generated or analysed during the current study.
