# Supplementary material for: Chinese visceral adiposity index and its transition patterns: impact on cardiovascular and cerebrovascular diseases in a national cohort study
Source: Lipids Health Dis. 2024 Apr 29;23:124. doi: 10.1186/s12944-024-02105-0 (PMC11057120; doi:10.1186/s12944-024-02105-0)
Supplement: Supplementary file 1 — Supplementary Material 1 [file 12944_2024_2105_MOESM1_ESM.docx]

**Supplementary material**

[**Figure S1. Distribution of participants’ CVAI in wave 2011(a) and wave 2015(b). 2**](#_Toc162446831)

[**Figure S2. The variance inflation factor (VIF) values for all variables in Model 4. 3**](#_Toc162446832)

[**Figure S3. Matrix diagram of the distribution of missing values 4**](#_Toc162446833)

[**Figure S4. The regional distribution of the study participants 5**](#_Toc162446834)

[**Figure S5. E-value analysis to evaluate the extent of unmeasured confounders in our model 4. 6**](#_Toc162446835)

[**Figure S6. Receiver operating characteristic curves of abdominal obesity indices for predicting cardiovascular and cerebrovascular diseases. 7**](#_Toc162446836)

[**Table S1. Definition of CVAI, LAP, and VAI. 8**](#_Toc162446837)

[**Table S2. The results of the Schoenfeld residual test. 9**](#_Toc162446838)

[**Table S3. Distribution of missing data. 10**](#_Toc162446839)

[**Table S4. Baseline characteristics of excluded and included participants. 11**](#_Toc162446840)

[**Table S5. Characteristics of participants stratified by baseline patterns and transition patterns of CVAI after multiple imputation. 12**](#_Toc162446841)

[**Table S6. Baseline characteristics of participants stratified by gender. 14**](#_Toc162446842)

[**Table S7. Baseline characteristics of participants stratified by outcome. 15**](#_Toc162446843)

[**Table S8. The association of CVAI with cardiovascular and cerebrovascular diseases using logistic regression. 17**](#_Toc162446844)

[**Table S9. The association of CVAI with cardiovascular and cerebrovascular diseases after further excluding individuals experienced cardiovascular and cerebrovascular diseases during wave 2. 18**](#_Toc162446845)

[**Table S10. The association of CVAI with cardiovascular and cerebrovascular diseases after excluding individuals with concomitant comorbidities. 19**](#_Toc162446846)

[**Table S11. The association of CVAI with cardiovascular and cerebrovascular diseases after excluding individuals with cancer in wave 2011. 20**](#_Toc162446847)

[**Table S12. Predictive performance of abdominal obesity indices for incident Cardiovascular and Cerebrovascular Diseases. 21**](#_Toc162446848)

# Figure S1. Distribution of participants’ CVAI in wave 2011(a) and wave 2015(b).


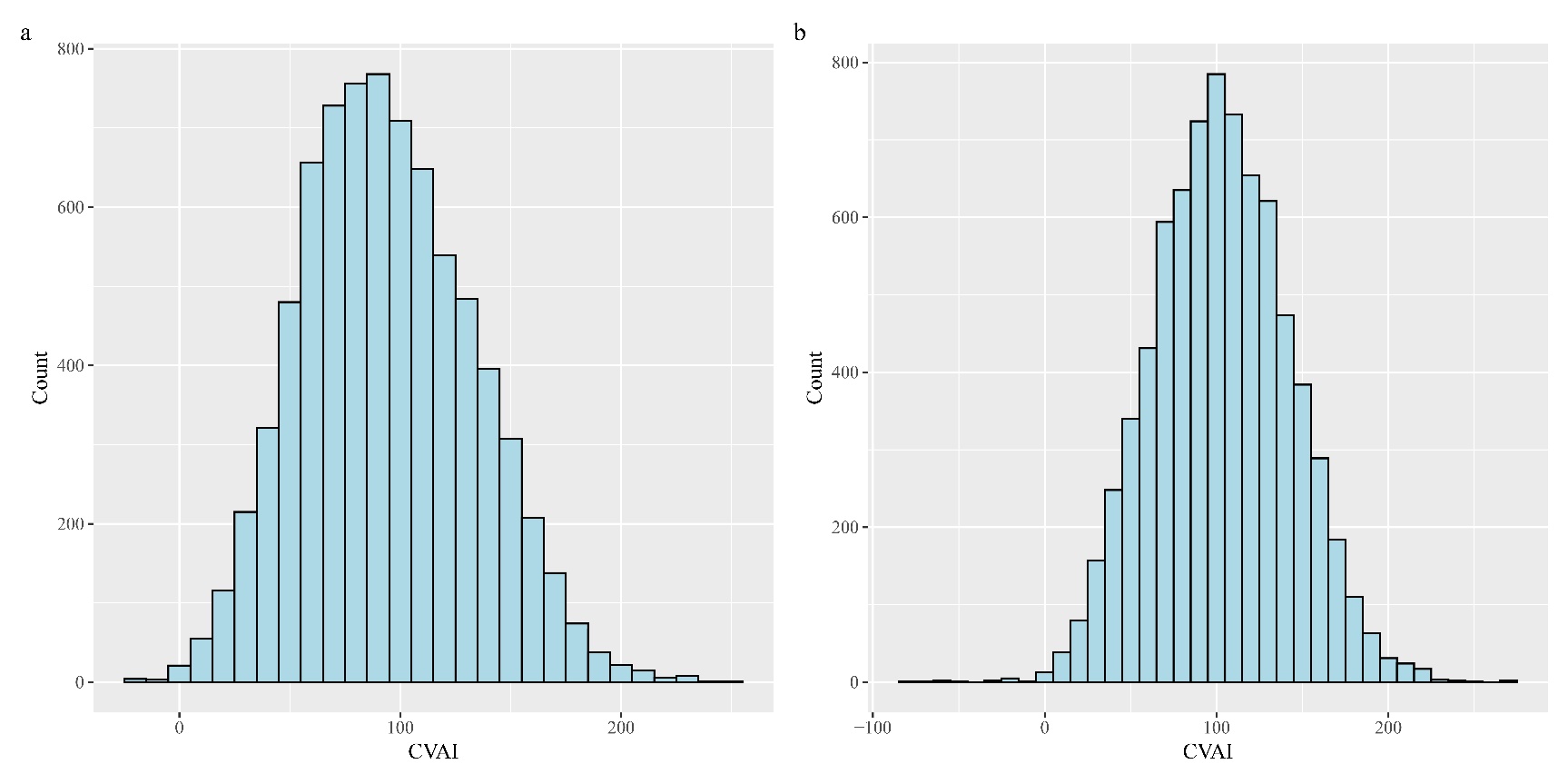


#
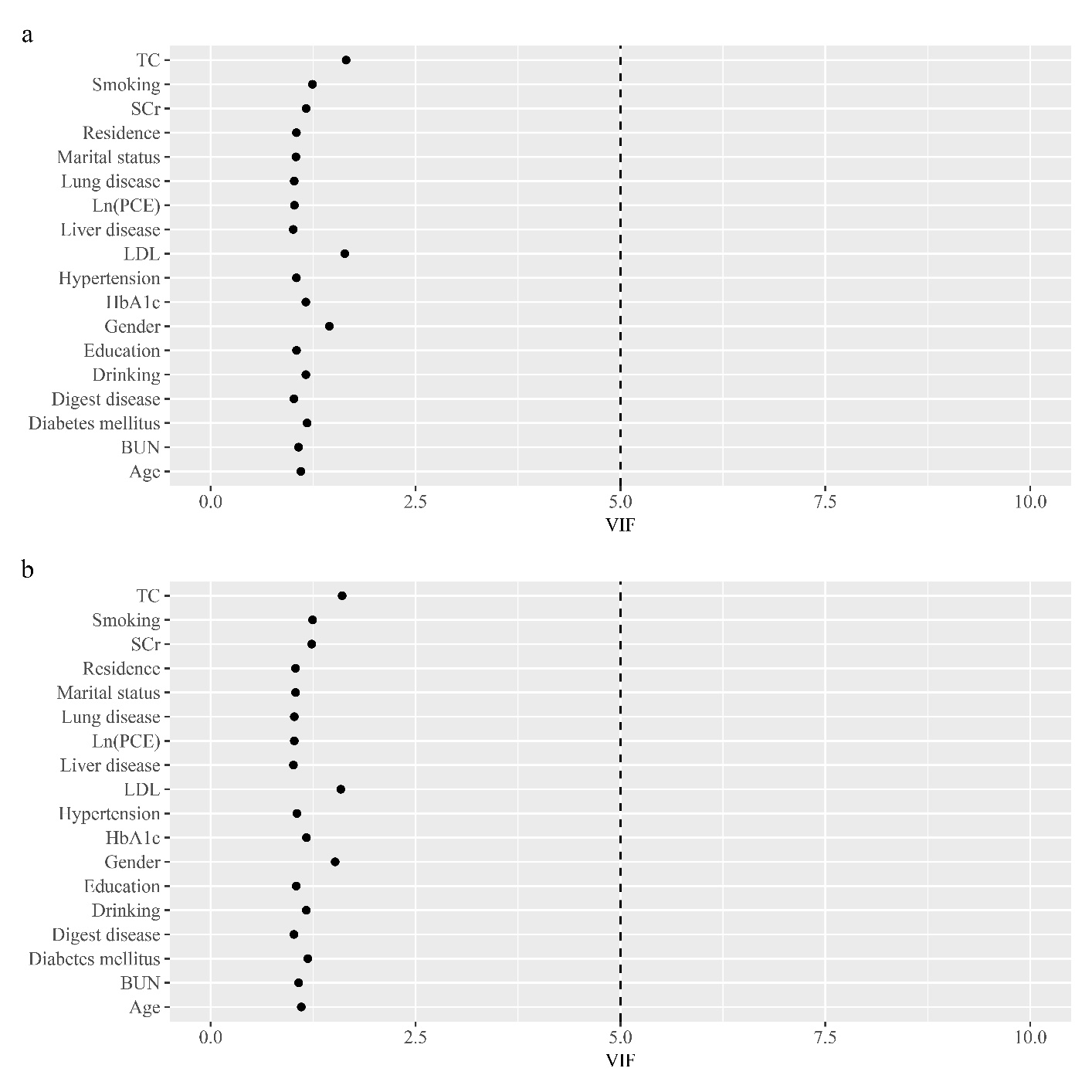
Figure S2. The variance inflation factor (VIF) values for all variables in Model 4.

1. The variance inflation factor (VIF) values for all variables in Model 4 of baseline patterns analysis
2. The variance inflation factor (VIF) values for all variables in Model 4 of transition patterns analysis

Abbreviations: PCE per capita expenditures, TC total cholesterol, LDL low-density lipoprotein, HbA1c glycosylated hemoglobin A1c, Scr serum creatinine, BUN blood urea nitrogen

# Figure S3. Matrix diagram of the distribution of missing values


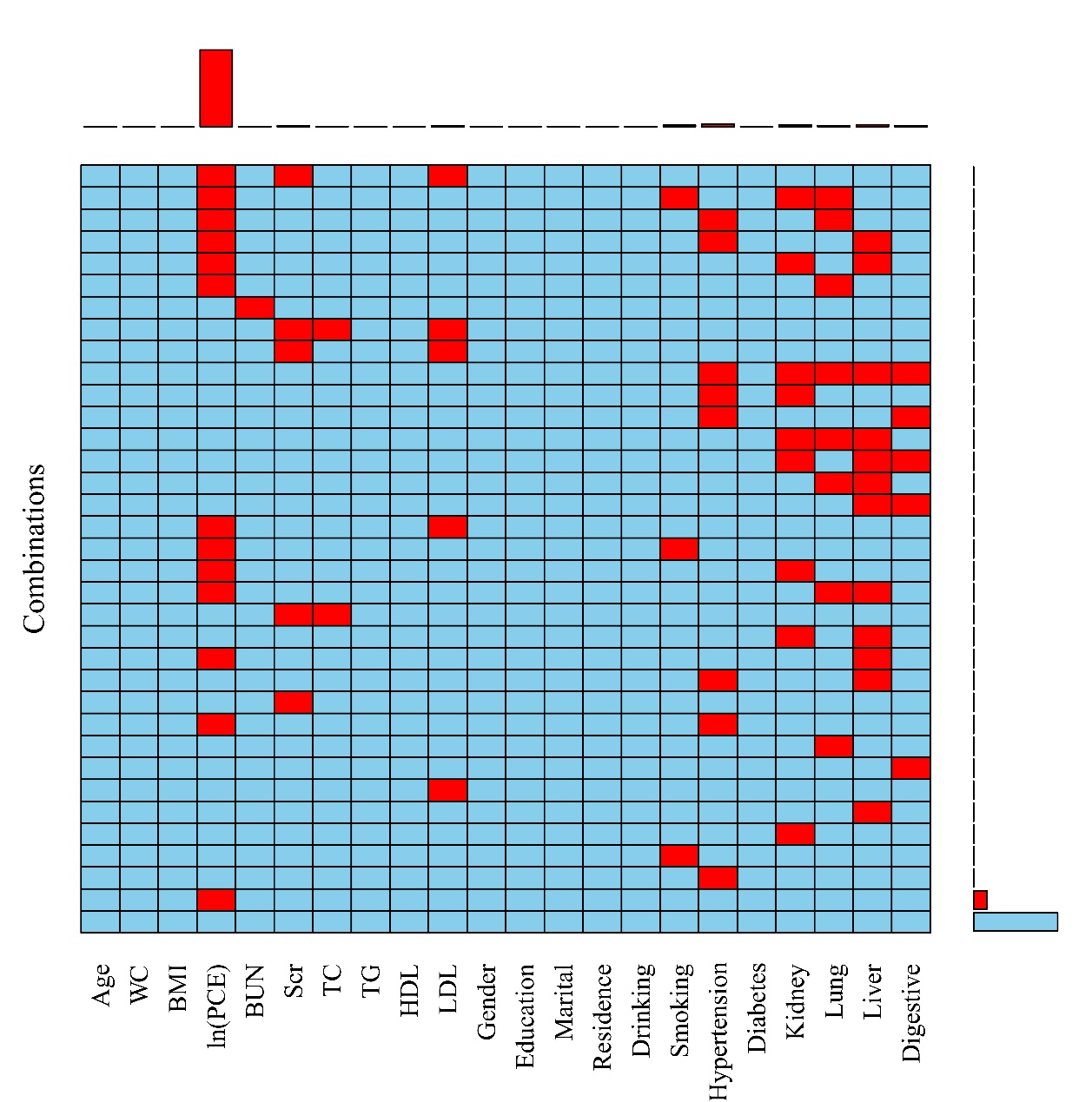


Abbreviations: PCE per capita expenditures, BMI body mass index, WC waist circumference, TC total cholesterol, TG triglycerides, HDL high-density lipoprotein, LDL low-density lipoprotein, HbA1c glycosylated hemoglobin A1c, Scr serum creatinine, BUN blood urea nitrogen

# Figure S4. The regional distribution of the study participants


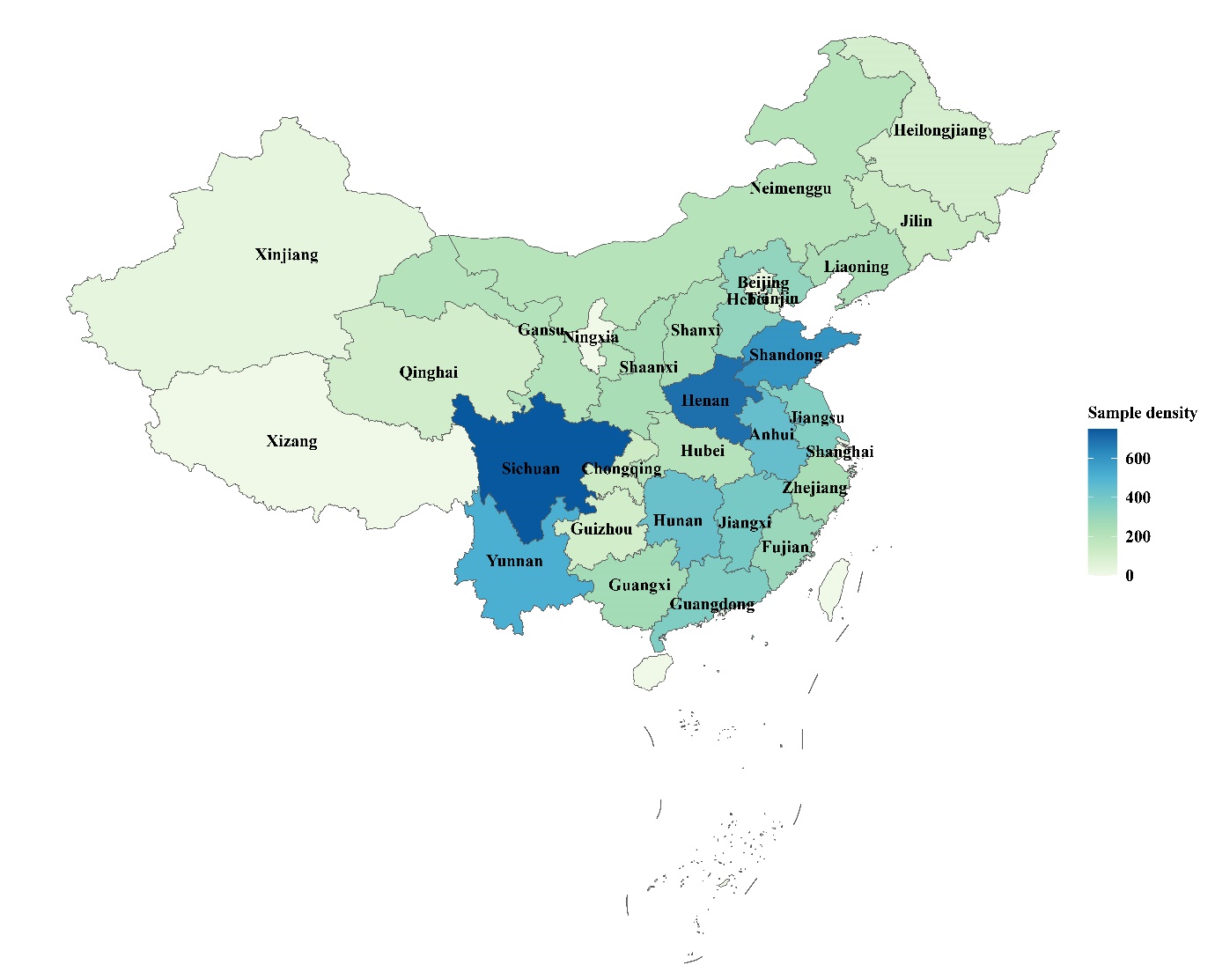


# Figure S5. E-value analysis to evaluate the extent of unmeasured confounders in our model 4.


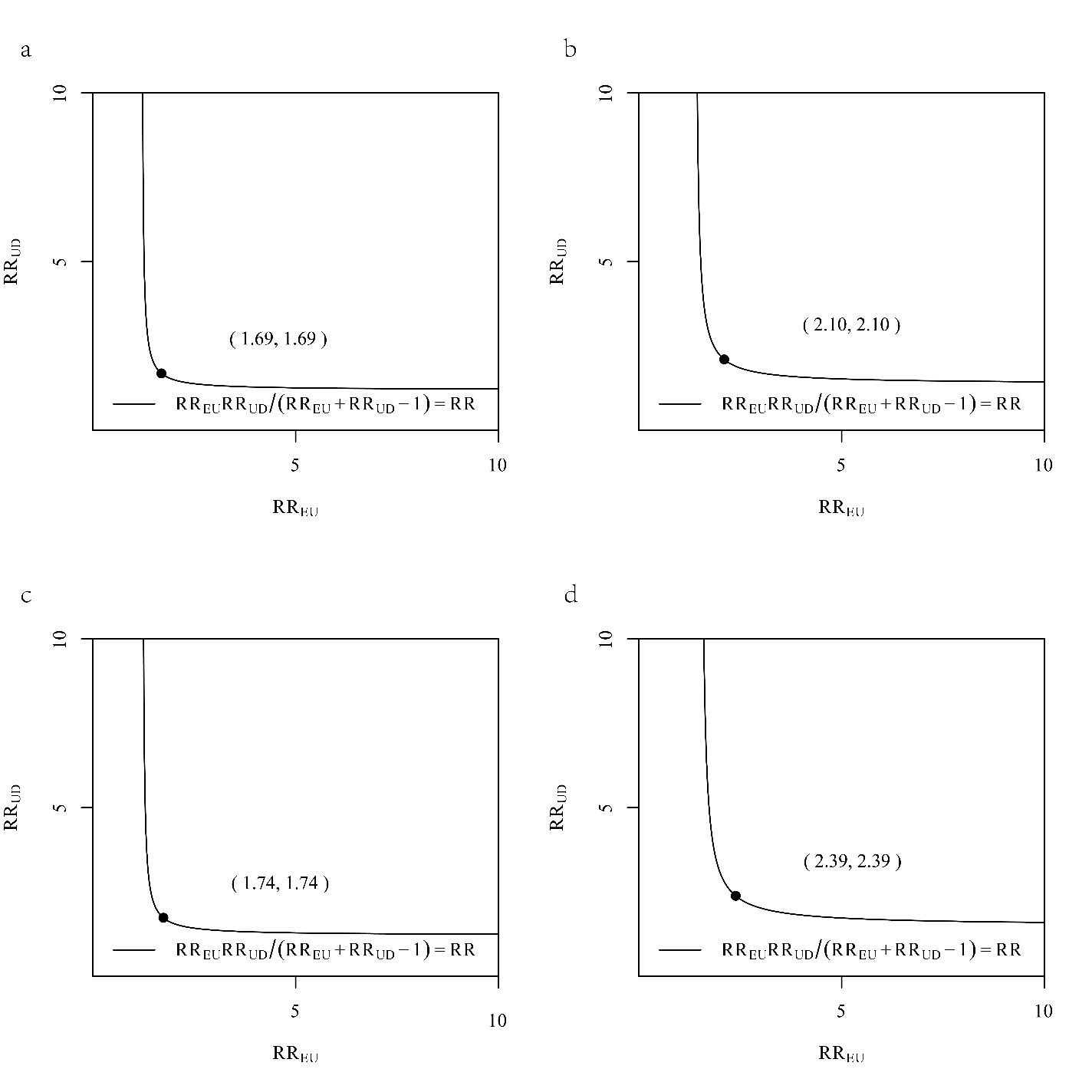


Notes: **(a)** Analysis of e-values for Per SD increment in model 4; **(b)** Analysis of e-values for the high baseline patterns in model 4; **(c)** Analysis of e-values for the low-high transition patterns in model 4; **(d)** Analysis of e-values for the high-high transition patterns in model 4

# Figure S6. Receiver operating characteristic curves of abdominal obesity indices for predicting cardiovascular and cerebrovascular diseases.


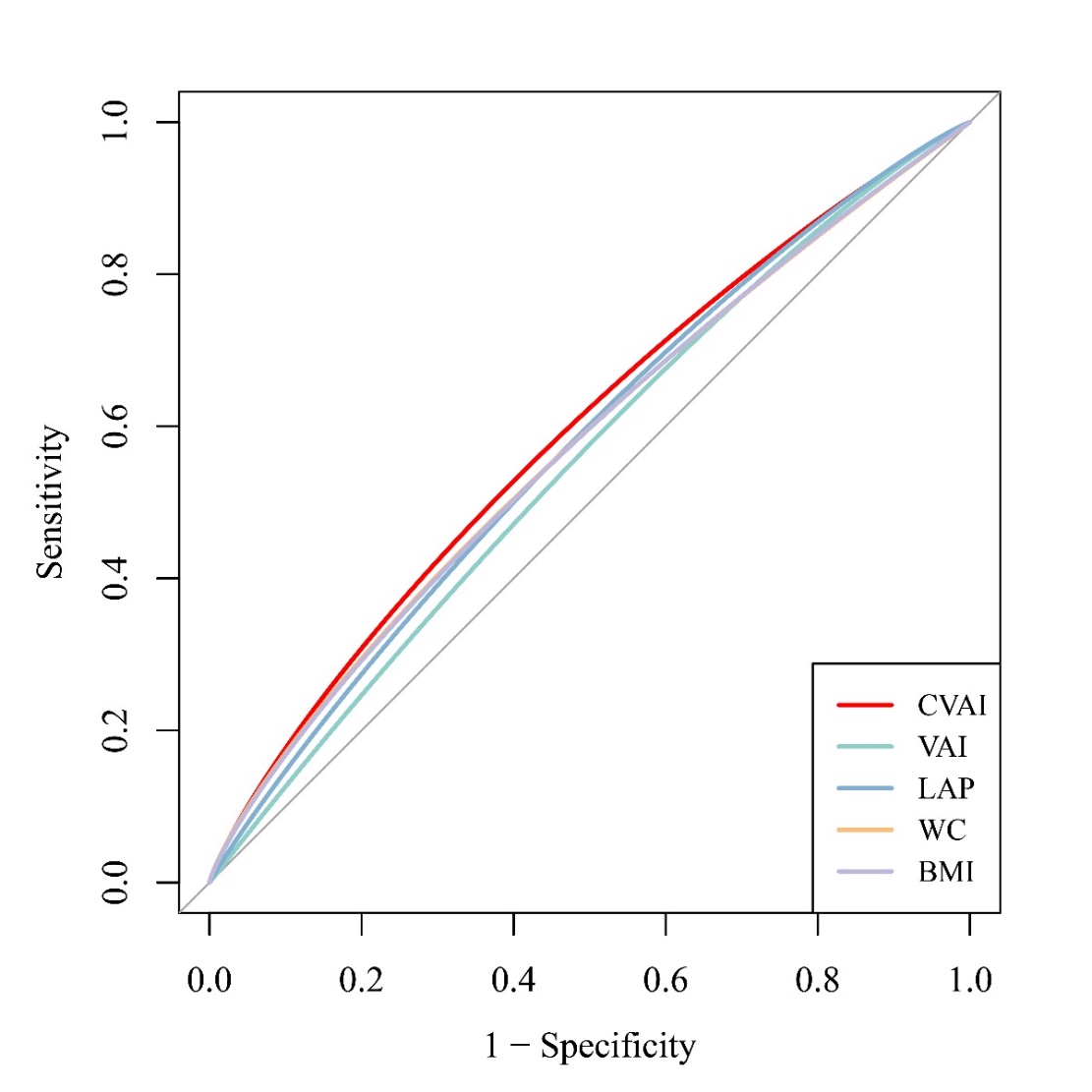


Abbreviations: CVAI, Chinese visceral adiposity index; VAI, visceral adiposity index; LAP, lipid accumulation product; WC, waist circumference; BMI, body mass index

# Table S1. Definition of CVAI, LAP, and VAI.

| **Variables** | **Formulas** |
| --- | --- |
| CVAI (male) | CVAI = -267.93 + 0.68 × age + 0.03 × BMI + 4.00 × WC + 22.00 × Lg (TG) - 16.32 × HDL |
| CVAI (female) | CVAI = -187.32 + 1.71 × age + 4.23 × BMI + 1.12 × WC + 39.76 × Lg (TG) - 11.66 × HDL |
| LAP (male) | LAP = (WC-65) × TG |
| LAP (female) | LAP = (WC-58) × TG |
| VAI (male) | VAI = [WC/(39.68 + 1.88×BMI)] × (TG/1.03) ×(1.31/HDL) |
| VAI (female) | VAI = [WC/(36.58 + 1.89×BMI)] × (TG/0.81) ×(1.52/HDL) |

BMI (kg/m^2^), WC (cm), age (years), TG (mmol/L), and HDL (mmol/L) are used to in the formulas above

BMI, body mass index; CI, confidence interval; CVAI, Chinese visceral adiposity index; HDL, high density lipoprotein; LAP, lipid accumulation product; TG, triglycerides; VAI, visceral adiposity index; WC, waist circumference

# Table S2. The results of the Schoenfeld residual test.

| **CVAI** | **Model1** | **Model2** | **Model3** | **Model4** |
| --- | --- | --- | --- | --- |
| Per SD increase | 0.84 | 0.22 | 0.24 | 0.06 |
| Baseline patterns | 0.97 | 0.13 | 0.34 | 0.20 |
| Transition patterns | 0.63 | 0.26 | 0.27 | 0.08 |

Model 1: unadjusted

Model 2: adjusted for age, gender, residence, marital status, educational level, ln(PCE)

Model 3: model 2 + further adjusted for smoking, alcohol consumption, hypertension, diabetes, lung disease, liver disease, digestive disease

Model 4: model 3 + further adjusted for TC, LDL, HbA1c, Scr, BUN

CI confidence interval, CVAI Chinese visceral adiposity index, PCE per capita expenditures, TC total cholesterol, LDL low-density lipoprotein, HbA1c glycosylated hemoglobin A1c, Scr serum creatinine, BUN blood urea nitrogen

# Table S3. Distribution of missing data.

| Characteristics | Number of missing values | Proportion of missing values(%) | Disposition |
| --- | --- | --- | --- |
| BUN | 1 | 0.01 | Multiple imputation |
| TC | 3 | 0.04 | Multiple imputation |
| Scr | 10 | 0.13 | Multiple imputation |
| Digestive | 12 | 0.16 | Multiple imputation |
| LDL | 14 | 0.18 | Multiple imputation |
| Lung | 14 | 0.18 | Multiple imputation |
| Smoking | 22 | 0.29 | Multiple imputation |
| Kidney | 22 | 0.29 | Multiple imputation |
| Liver | 28 | 0.36 | Multiple imputation |
| Hypertension | 34 | 0.44 | Multiple imputation |
| Ln (PCE) | 1059 | 13.72 | Multiple imputation |

Abbreviations: PCE per capita expenditures, TC total cholesterol, LDL low-density lipoprotein, Scr serum creatinine, BUN blood urea nitrogen

# Table S4. Baseline characteristics of excluded and included participants.

|  | **Overall** | **Excluded** | **Included** | ***P* value** |
| --- | --- | --- | --- | --- |
| n | 17,708 | 9,991 | 7,717 |  |
| Age, years | 58.50 ± 10.17 | 58.24 ± 10.80 | 58.82 ± 9.31 | <0.001 |
| Gender, n (%) |  |  |  | 0.475 |
| Male | 8478 (47.9) | 4807 (48.1) | 3671 (47.6) |  |
| Female | 9228 (52.1) | 5182 (51.9) | 4046 (52.4) |  |
| Residence, n (%) |  |  |  | <0.001 |
| Urban | 7171 (40.5) | 4578 (45.8) | 2593 (33.6) |  |
| Rural | 10537 (59.5) | 5413 (54.2) | 5124 (66.4) |  |
| Marital, n (%) |  |  |  | <0.001 |
| Married and cohabiting | 14170 (80.1) | 7707 (77.2) | 6463 (83.8) |  |
| Single | 3526 (19.9) | 2272 (22.8) | 1254 (16.2) |  |
| Ln(PCE), n (%) |  |  |  | <0.001 |
| Bottom tertile | 4918 (33.3) | 2528 (31.2) | 2390 (35.9) |  |
| Middle tertile | 4922 (33.3) | 2605 (32.1) | 2317 (34.8) |  |
| Top tertile | 4922 (33.3) | 2972 (36.7) | 1950 (29.3) |  |
| Education, n (%) |  |  |  | <0.001 |
| Middle School or below | 15545 (87.9) | 8532 (85.5) | 7013 (90.9) |  |
| High school or above | 2147 (12.1) | 1443 (14.5) | 704 (9.1) |  |
| Alcohol drinking, n (%) |  |  |  | 0.004 |
| Non-drinker | 11785 (67.1) | 6693 (68.1) | 5092 (66.0) |  |
| Drinker | 5767 (32.9) | 3142 (31.9) | 2625 (34.0) |  |
| Smoking, n (%) |  |  |  | <0.001 |
| Non-smoker | 12036 (71.2) | 6765 (73.4) | 5271 (68.5) |  |
| smoker | 4871 (28.8) | 2447 (26.6) | 2424 (31.5) |  |
| Hypertension, n (%) | 4783 (27.4) | 2971 (30.4) | 1812 (23.6) | <0.001 |
| Diabetes mellitus, n (%) | 2381 (20.2) | 1141 (27.3) | 1240 (16.3) | <0.001 |
| Lung disease, n (%) | 2065 (11.8) | 1230 (12.6) | 835 (10.8) | <0.001 |
| Liver disease, n (%) | 708 (4.1) | 447 (4.6) | 261 (3.4) | <0.001 |
| Digestive disease, n (%) | 4336 (24.8) | 2433 (24.8) | 1903 (24.7) | 0.884 |
| BMI, kg/m2 | 24.16 ± 35.80 | 25.32 ± 54.18 | 23.26 ± 3.53 | 0.001 |
| WC, cm | 84.28 ± 12.62 | 83.60 ± 15.42 | 84.81 ± 9.86 | <0.001 |
| TC, mg/dL | 192.97 ± 38.89 | 191.71 ± 39.45 | 193.61 ± 38.59 | 0.013 |
| TG, mg/dL | 134.91 ± 110.26 | 142.09 ± 114.15 | 131.24 ± 108.05 | <0.001 |
| HDL, mg/dL | 50.84 ± 15.33 | 49.42 ± 15.16 | 51.57 ± 15.36 | <0.001 |
| LDL, mg/dL | 115.99 ± 34.91 | 115.14 ± 34.96 | 116.43 ± 34.88 | 0.059 |
| HbA1c, % | 5.26 ± 0.82 | 5.27 ± 0.86 | 5.26 ± 0.80 | 0.522 |
| Scr, mg/dL | 0.78 ± 0.24 | 0.79 ± 0.23 | 0.78 ± 0.24 | 0.196 |
| BUN, mg/dL | 15.75 ± 4.65 | 15.72 ± 4.78 | 15.76 ± 4.58 | 0.624 |

Mean ± SD for continuous variables and counts (percentages) for categorical variables.

PCE per capita expenditures, BMI body mass index, WC waist circumference, TC total cholesterol, TG triglycerides, HDL high-density lipoprotein, LDL low-density lipoprotein, HbA1c glycosylated hemoglobin A1c, Scr serum creatinine, BUN blood urea nitrogen

# Table S5. Characteristics of participants stratified by baseline patterns and transition patterns of CVAI after multiple imputation.

|  | **Baseline patterns** | | | | **Transition patterns** | | | | | |
| --- | --- | --- | --- | --- | --- | --- | --- | --- | --- | --- |
|  | **Overall** | **Low** | **High** | ***P* Value** | **Overall** | **Low-Low** | **Low-High** | **High-Low** | **High-High** | ***P* Value** |
| n | 7,717 | 3,995 | 3,722 |  | 4,993 | 1,859 | 696 | 183 | 2,255 |  |
| Baseline CVAI | 94.04 ± 38.87 | 64.09 ± 21.15 | 126.18 ± 25.62 | <0.001 | 94.71 ± 38.50 | 59.89 ± 21.32 | 77.85 ± 13.70 | 111.38 ± 18.56 | 127.26 ± 25.53 | <0.001 |
| Age, years | 58.82 ± 9.31 | 57.49 ± 8.87 | 60.24 ± 9.56 | <0.001 | 58.33 ± 8.65 | 57.45 ± 8.45 | 56.51 ± 8.21 | 58.52 ± 9.15 | 59.61 ± 8.73 | <0.001 |
| Gender, n (%) |  |  |  | <0.001 |  |  |  |  |  | <0.001 |
| Male | 3671 (47.6) | 2276 (57.0) | 1395 (37.5) |  | 2335 (46.8) | 1172 (63.0) | 270 (38.8) | 108 (59.0) | 785 (34.8) |  |
| Female | 4046 (52.4) | 1719 (43.0) | 2327 (62.5) |  | 2658 (53.2) | 687 (37.0) | 426 (61.2) | 75 (41.0) | 1470 (65.2) |  |
| Residence, n (%) |  |  |  | <0.001 |  |  |  |  |  | <0.001 |
| Urban | 2593 (33.6) | 1097 (27.5) | 1496 (40.2) |  | 1615 (32.3) | 491 (26.4) | 191 (27.4) | 57 (31.1) | 876 (38.8) |  |
| Rural | 5124 (66.4) | 2898 (72.5) | 2226 (59.8) |  | 3378 (67.7) | 1368 (73.6) | 505 (72.6) | 126 (68.9) | 1379 (61.2) |  |
| Marital, n (%) |  |  |  | 0.044 |  |  |  |  |  | 0.871 |
| Married and cohabiting | 6463 (83.8) | 3379 (84.6) | 3084 (82.9) |  | 4289 (85.9) | 1602 (86.2) | 598 (85.9) | 160 (87.4) | 1929 (85.5) |  |
| Single | 1254 (16.2) | 616 (15.4) | 638 (17.1) |  | 704 (14.1) | 257 (13.8) | 98 (14.1) | 23 (12.6) | 326 (14.5) |  |
| Ln(PCE), n (%) |  |  |  | <0.001 |  |  |  |  |  | 0.002 |
| Bottom tertile | 2573 (33.3) | 1460 (36.5) | 1113 (29.9) |  | 1757 (35.2) | 724 (38.9) | 237 (34.1) | 58 (31.7) | 738 (32.7) |  |
| Middle tertile | 2574 (33.4) | 1320 (33.0) | 1254 (33.7) |  | 1657 (33.2) | 592 (31.8) | 236 (33.9) | 70 (38.3) | 759 (33.7) |  |
| Top tertile | 2570 (33.3) | 1215 (30.4) | 1355 (36.4) |  | 1579 (31.6) | 543 (29.2) | 223 (32.0) | 55 (30.1) | 758 (33.6) |  |
| Education, n (%) |  |  |  | 0.097 |  |  |  |  |  | 0.546 |
| Middle School or below | 7013 (90.9) | 3652 (91.4) | 3361 (90.3) |  | 4571 (91.5) | 1713 (92.1) | 633 (90.9) | 164 (89.6) | 2061 (91.4) |  |
| High school or above | 704 (9.1) | 343 (8.6) | 361 (9.7) |  | 422 (8.5) | 146 (7.9) | 63 (9.1) | 19 (10.4) | 194 (8.6) |  |
| Alcohol drinking, n (%) |  |  |  | <0.001 |  |  |  |  |  | <0.001 |
| Non-drinker | 5092 (66.0) | 2418 (60.5) | 2674 (71.8) |  | 3319 (66.5) | 1084 (58.3) | 484 (69.5) | 111 (60.7) | 1640 (72.7) |  |
| Drinker | 2625 (34.0) | 1577 (39.5) | 1048 (28.2) |  | 1674 (33.5) | 775 (41.7) | 212 (30.5) | 72 (39.3) | 615 (27.3) |  |
| Smoking, n (%) |  |  |  | <0.001 |  |  |  |  |  | <0.001 |
| Non-smoker | 5279 (68.4) | 2403 (60.2) | 2876 (77.3) |  | 3450 (69.1) | 1045 (56.2) | 506 (72.7) | 117 (63.9) | 1782 (79.0) |  |
| smoker | 2438 (31.6) | 1592 (39.8) | 846 (22.7) |  | 1543 (30.9) | 814 (43.8) | 190 (27.3) | 66 (36.1) | 473 (21.0) |  |
| Hypertension, n (%) | 1817 (23.5) | 558 (14.0) | 1259 (33.8) | <0.001 | 1194 (23.9) | 225 (12.1) | 133 (19.1) | 36 (19.7) | 800 (35.5) | <0.001 |
| Diabetes mellitus, n (%) | 1242 (16.1) | 419 (10.5) | 823 (22.1) | <0.001 | 791 (15.8) | 194 (10.4) | 61 (8.8) | 48 (26.2) | 488 (21.6) | <0.001 |
| Lung disease, n (%) | 836 (10.8) | 471 (11.8) | 365 (9.8) | 0.006 | 508 (10.2) | 212 (11.4) | 72 (10.3) | 19 (10.4) | 205 (9.1) | 0.111 |
| Liver disease, n (%) | 261 (3.4) | 151 (3.8) | 110 (3.0) | 0.053 | 156 (3.1) | 62 (3.3) | 29 (4.2) | 8 (4.4) | 57 (2.5) | 0.095 |
| Digestive disease, n (%) | 1906 (24.7) | 1088 (27.2) | 818 (22.0) | <0.001 | 1179 (23.6) | 493 (26.5) | 167 (24.0) | 44 (24.0) | 475 (21.1) | 0.001 |
| BMI, kg/m2 | 23.26 ± 3.53 | 21.13 ± 2.37 | 25.56 ± 3.11 | <0.001 | 23.41 ± 3.50 | 20.74 ± 2.15 | 22.59 ± 2.15 | 23.68 ± 2.49 | 25.85 ± 3.04 | <0.001 |
| WC, cm | 84.81 ± 9.86 | 78.04 ± 6.20 | 92.07 ± 7.66 | <0.001 | 85.13 ± 9.76 | 77.18 ± 5.90 | 81.30 ± 5.76 | 88.52 ± 5.50 | 92.58 ± 7.50 | <0.001 |
| TC, mg/dL | 193.61 ± 38.59 | 188.22 ± 36.49 | 199.39 ± 39.93 | <0.001 | 193.55 ± 38.38 | 186.46 ± 35.51 | 192.18 ± 35.72 | 192.92 ± 39.66 | 199.86 ± 40.27 | <0.001 |
| TG, mg/dL | 131.24 ± 108.05 | 97.04 ± 60.79 | 167.95 ± 132.81 | <0.001 | 133.20 ± 108.50 | 94.78 ± 54.39 | 102.18 ± 54.09 | 152.93 ± 84.49 | 172.85 ± 137.98 | <0.001 |
| HDL, mg/dL | 51.57 ± 15.36 | 57.41 ± 15.44 | 45.30 ± 12.55 | <0.001 | 51.21 ± 15.32 | 57.94 ± 16.04 | 54.86 ± 13.50 | 46.48 ± 13.15 | 44.91 ± 12.43 | <0.001 |
| LDL, mg/dL | 116.33 ± 35.02 | 113.03 ± 32.16 | 119.87 ± 37.52 | <0.001 | 116.10 ± 34.83 | 111.02 ± 31.52 | 118.80 ± 31.03 | 115.65 ± 36.51 | 119.50 ± 37.78 | <0.001 |
| HbA1c, % | 5.26 ± 0.80 | 5.15 ± 0.68 | 5.37 ± 0.90 | <0.001 | 5.25 ± 0.80 | 5.14 ± 0.68 | 5.15 ± 0.59 | 5.30 ± 0.93 | 5.37 ± 0.90 | <0.001 |
| Scr, mg/dL | 0.78 ± 0.24 | 0.78 ± 0.20 | 0.78 ± 0.27 | 0.867 | 0.77 ± 0.18 | 0.78 ± 0.17 | 0.75 ± 0.19 | 0.80 ± 0.20 | 0.76 ± 0.18 | <0.001 |
| BUN, mg/dL | 15.76 ± 4.58 | 15.95 ± 4.75 | 15.56 ± 4.38 | <0.001 | 15.64 ± 4.33 | 16.00 ± 4.45 | 15.55 ± 4.48 | 15.13 ± 4.18 | 15.42 ± 4.17 | <0.001 |
| Mean ± SD for continuous variables and counts (percentages) for categorical variables. CVAI Chinese visceral adiposity index, PCE per capita expenditures, BMI body mass index, WC waist circumference, TC total cholesterol, TG triglycerides, HDL high-density lipoprotein, LDL low-density lipoprotein, HbA1c glycosylated hemoglobin A1c, Scr serum creatinine, BUN blood urea nitrogen High group: CVAI ≥ 88.42 (females), ≥ 101.80 (males); Low group: CVAI < 88.42 (females), < 101.80 (males) | | | | | | | | | | |

# Table S6. Baseline characteristics of participants stratified by gender.

|  | **Overall** | **Male** | **Female** | ***P* value** |
| --- | --- | --- | --- | --- |
| n | 7,717 | 3,671 | 4,046 |  |
| Baseline CVAI | 94.04 ± 38.87 | 91.20 ± 43.24 | 96.61 ± 34.24 | <0.001 |
| Age, years | 58.82 ± 9.31 | 59.47 ± 9.17 | 58.22 ± 9.39 | <0.001 |
| Residence, n (%) |  |  |  | 0.162 |
| Urban | 2593 (33.6) | 1204 (32.8) | 1389 (34.3) |  |
| Rural | 5124 (66.4) | 2467 (67.2) | 2657 (65.7) |  |
| Marital, n (%) |  |  |  | <0.001 |
| Married and cohabiting | 6463 (83.8) | 3231 (88.0) | 3232 (79.9) |  |
| Single | 1254 (16.2) | 440 (12.0) | 814 (20.1) |  |
| Ln(PCE), n (%) |  |  |  | 0.357 |
| Bottom tertile | 2573 (33.3) | 1206 (32.9) | 1367 (33.8) |  |
| Middle tertile | 2574 (33.4) | 1213 (33.0) | 1361 (33.6) |  |
| Top tertile | 2570 (33.3) | 1252 (34.1) | 1318 (32.6) |  |
| Education, n (%) |  |  |  | <0.001 |
| Middle School or below | 7013 (90.9) | 3214 (87.6) | 3799 (93.9) |  |
| High school or above | 704 (9.1) | 457 (12.4) | 247 (6.1) |  |
| Alcohol drinking, n (%) |  |  |  | <0.001 |
| Non-drinker | 5092 (66.0) | 1554 (42.3) | 3538 (87.4) |  |
| Drinker | 2625 (34.0) | 2117 (57.7) | 508 (12.6) |  |
| Smoking, n (%) |  |  |  | <0.001 |
| Non-smoker | 5279 (68.4) | 1456 (39.7) | 3823 (94.5) |  |
| smoker | 2438 (31.6) | 2215 (60.3) | 223 (5.5) |  |
| Hypertension, n (%) | 1817 (23.5) | 805 (21.9) | 1012 (25.0) | 0.002 |
| Diabetes mellitus, n (%) | 1242 (16.1) | 596 (16.2) | 646 (16.0) | 0.772 |
| Lung disease, n (%) | 836 (10.8) | 484 (13.2) | 352 (8.7) | <0.001 |
| Liver disease, n (%) | 261 (3.4) | 130 (3.5) | 131 (3.2) | 0.501 |
| Digestive disease, n (%) | 1906 (24.7) | 825 (22.5) | 1081 (26.7) |  |
| BMI, kg/m2 | 23.26 ± 3.53 | 22.76 ± 3.32 | 23.72 ± 3.66 | <0.001 |
| WC, cm | 84.81 ± 9.86 | 84.50 ± 9.56 | 85.09 ± 10.12 | 0.009 |
| TC, mg/dL | 193.61 ± 38.59 | 188.32 ± 38.12 | 198.41 ± 38.39 | <0.001 |
| TG, mg/dL | 131.24 ± 108.05 | 127.31 ± 116.77 | 134.82 ± 99.35 | 0.002 |
| HDL, mg/dL | 51.57 ± 15.36 | 50.99 ± 16.18 | 52.10 ± 14.56 | 0.002 |
| LDL, mg/dL | 116.33 ± 35.02 | 112.05 ± 34.42 | 120.21 ± 35.10 | <0.001 |
| HbA1c, % | 5.26 ± 0.80 | 5.23 ± 0.77 | 5.28 ± 0.83 | 0.006 |
| Scr, mg/dL | 0.78 ± 0.24 | 0.88 ± 0.28 | 0.69 ± 0.15 | <0.001 |
| BUN, mg/dL | 15.76 ± 4.58 | 16.53 ± 4.71 | 15.07 ± 4.34 | <0.001 |

Mean ± SD for continuous variables and counts (percentages) for categorical variables.

CVAI Chinese visceral adiposity index, PCE per capita expenditures, BMI body mass index, WC waist circumference, TC total cholesterol, TG triglycerides, HDL high-density lipoprotein, LDL low-density lipoprotein, HbA1c glycosylated hemoglobin A1c, Scr serum creatinine, BUN blood urea nitrogen

# Table S7. Baseline characteristics of participants stratified by outcome.

|  | **Overall** | **Without outcome** | **With outcome** | **P value** |
| --- | --- | --- | --- | --- |
| n | 7,717 | 5,956 | 1,761 |  |
| Baseline CVAI | 94.04 ± 38.87 | 91.16 ± 38.09 | 103.76 ± 39.90 | <0.001 |
| Age, years | 58.82 ± 9.31 | 58.48 ± 9.45 | 59.95 ± 8.71 | <0.001 |
| Gender, n (%) |  |  |  | <0.001 |
| Male | 3671 (47.6) | 2922 (49.1) | 749 (42.5) |  |
| Female | 4046 (52.4) | 3034 (50.9) | 1012 (57.5) |  |
| Residence, n (%) |  |  |  | 1.000 |
| Urban | 2593 (33.6) | 2001 (33.6) | 592 (33.6) |  |
| Rural | 5124 (66.4) | 3955 (66.4) | 1169 (66.4) |  |
| Marital, n (%) |  |  |  | 0.788 |
| Married and cohabiting | 6463 (83.8) | 4984 (83.7) | 1479 (84.0) |  |
| Single | 1254 (16.2) | 972 (16.3) | 282 (16.0) |  |
| Ln(PCE), n (%) |  |  |  | 0.551 |
| Bottom tertile | 2573 (33.3) | 2003 (33.6) | 570 (32.4) |  |
| Middle tertile | 2574 (33.4) | 1971 (33.1) | 603 (34.2) |  |
| Top tertile | 2570 (33.3) | 1982 (33.3) | 588 (33.4) |  |
| Education, n (%) |  |  |  | 0.788 |
| Middle School or below | 7013 (90.9) | 5416 (90.9) | 1597 (90.7) |  |
| High school or above | 704 (9.1) | 540 (9.1) | 164 (9.3) |  |
| Alcohol drinking, n (%) |  |  |  | <0.001 |
| Non-drinker | 5092 (66.0) | 3863 (64.9) | 1229 (69.8) |  |
| Drinker | 2625 (34.0) | 2093 (35.1) | 532 (30.2) |  |
| Smoking, n (%) |  |  |  | 0.001 |
| Non-smoker | 5279 (68.4) | 4018 (67.5) | 1261 (71.6) |  |
| smoker | 2438 (31.6) | 1938 (32.5) | 500 (28.4) |  |
| Hypertension, n (%) | 1817 (23.5) | 1203 (20.2) | 614 (34.9) | <0.001 |
| Diabetes mellitus, n (%) | 1242 (16.1) | 916 (15.4) | 326 (18.5) | 0.002 |
| Lung disease, n (%) | 836 (10.8) | 571 (9.6) | 265 (15.0) | <0.001 |
| Liver disease, n (%) | 261 (3.4) | 181 (3.0) | 80 (4.5) | 0.003 |
| Digestive disease, n (%) | 1906 (24.7) | 1401 (23.5) | 505 (28.7) | <0.001 |
| BMI, kg/m2 | 23.26 ± 3.53 | 23.05 ± 3.44 | 23.99 ± 3.75 | <0.001 |
| WC, cm | 84.81 ± 9.86 | 84.16 ± 9.60 | 86.98 ± 10.41 | <0.001 |
| TC, mg/dL | 193.61 ± 38.59 | 192.57 ± 38.61 | 197.10 ± 38.34 | <0.001 |
| TG, mg/dL | 131.24 ± 108.05 | 129.04 ± 106.90 | 138.72 ± 111.55 | 0.001 |
| HDL, mg/dL | 51.57 ± 15.36 | 51.86 ± 15.41 | 50.60 ± 15.15 | 0.002 |
| LDL, mg/dL | 116.33 ± 35.02 | 115.43 ± 34.76 | 119.35 ± 35.70 | <0.001 |
| HbA1c, % | 5.26 ± 0.80 | 5.23 ± 0.76 | 5.34 ± 0.92 | <0.001 |
| Scr, mg/dL | 0.78 ± 0.24 | 0.78 ± 0.25 | 0.78 ± 0.19 | 0.398 |
| BUN, mg/dL | 15.76 ± 4.58 | 15.81 ± 4.62 | 15.61 ± 4.43 | 0.113 |

Mean ± SD for continuous variables and counts (percentages) for categorical variables.

CVAI Chinese visceral adiposity index, PCE per capita expenditures, BMI body mass index, WC waist circumference, TC total cholesterol, TG triglycerides, HDL high-density lipoprotein, LDL low-density lipoprotein, HbA1c glycosylated hemoglobin A1c, Scr serum creatinine, BUN blood urea nitrogen

# Table S8. The association of CVAI with cardiovascular and cerebrovascular diseases using logistic regression.

| **CVAI^a^** | **Total N** | **No. of events (Incident rate^b^)** | **Model 1** |  | **Model 2** |  | **Model 3** |  | **Model 4** |  |
| --- | --- | --- | --- | --- | --- | --- | --- | --- | --- | --- |
|  |  |  | **OR (95% CI)** | ***P* value** | **OR (95% CI)** | ***P* value** | **OR (95% CI)** | ***P* value** | **OR (95% CI)** | ***P* value** |
| Continues |  |  |  |  |  |  |  |  |  |  |
| Per SD increase | 7717 | 1761 (22.82) | 1.38(1.31-1.46) | <0.001 | 1.35(1.28-1.43) | <0.001 | 1.29(1.21-1.37) | <0.001 | 1.28(1.20-1.36) | <0.001 |
| Baseline patterns |  |  |  |  |  |  |  |  |  |  |
| Low | 3995 | 711 (17.80) | Ref. |  | Ref. |  | Ref. |  | Ref. |  |
| High | 3722 | 1050 (28.21) | 1.82(1.63-2.02) | <0.001 | 1.69(1.51-1.89) | <0.001 | 1.56(1.38-1.76) | <0.001 | 1.52(1.35-1.72) | <0.001 |
| Transition patterns |  |  |  |  |  |  |  |  |  |  |
| Low - Low | 1859 | 335 (18.02) | Ref. |  | Ref. |  | Ref. |  | Ref. |  |
| Low - High | 696 | 161 (23.13) | 1.37(1.11-1.69) | 0.004 | 1.33(1.07-1.65) | 0.009 | 1.29(1.03-1.60) | 0.025 | 1.26(1.01-1.57) | 0.036 |
| High - Low | 183 | 46 (25.14) | 1.53(1.06-2.16) | 0.019 | 1.47(1.02-2.09) | 0.034 | 1.41(0.97-2.02) | 0.062 | 1.37(0.95-1.96) | 0.087 |
| High - High | 2255 | 719 (31.88) | 2.13(1.84-2.47) | <0.001 | 1.94(1.66-2.27) | <0.001 | 1.74(1.48-2.05) | <0.001 | 1.67(1.42-1.98) | <0.001 |

Model 1: unadjusted

Model 2: adjusted for age, gender, residence, marital status, educational level, ln(PCE)

Model 3: model 2 + further adjusted for smoking, alcohol consumption, hypertension, diabetes, lung disease, liver disease, digestive disease

Model 4: model 3 + further adjusted for TC, LDL, HbA1c, Scr, BUN

OR odds ratio, CI confidence interval, CVAI Chinese visceral adiposity index, PCE per capita expenditures, TC total cholesterol, LDL low-density lipoprotein, HbA1c glycosylated hemoglobin A1c, Scr serum creatinine, BUN blood urea nitrogen

^a^ High group: CVAI ≥ 88.42 (females), ≥ 101.80 (males); Low group: CVAI < 88.42 (females), < 101.80 (males)

^b^ Incident rate was presented as a percentage

# Table S9. The association of CVAI with cardiovascular and cerebrovascular diseases after further excluding individuals experienced cardiovascular and cerebrovascular diseases during wave 2.

| **CVAI^a^** | **Total N** | **No. of events (Incident rate^b^)** | **Model 1** |  | **Model 2** |  | **Model 3** |  | **Model 4** |  |
| --- | --- | --- | --- | --- | --- | --- | --- | --- | --- | --- |
|  |  |  | **HR (95% CI)** | ***P* value** | **HR (95% CI)** | ***P* value** | **HR (95% CI)** | ***P* value** | **HR (95% CI)** | ***P* value** |
| Continues |  |  |  |  |  |  |  |  |  |  |
| Per SD increase | 6726 | 1376 (20.46) | 1.31 (1.24-1.38) | <0.001 | 1.27 (1.20-1.34) | <0.001 | 1.21 (1.14-1.28) | <0.001 | 1.19 (1.12-1.26) | <0.001 |
| Baseline patterns |  |  |  |  |  |  |  |  |  |  |
| Low | 3516 | 564 (16.04) | Ref. |  | Ref. |  | Ref. |  | Ref. |  |
| High | 3210 | 812 (25.3) | 1.65 (1.48-1.84) | <0.001 | 1.53 (1.37-1.71) | <0.001 | 1.41 (1.25-1.59) | <0.001 | 1.37 (1.22-1.54) | <0.001 |
| Transition patterns |  |  |  |  |  |  |  |  |  |  |
| Low - Low | 1737 | 269 (15.49) | Ref. |  | Ref. |  | Ref. |  | Ref. |  |
| Low - High | 642 | 137 (21.34) | 1.43 (1.16-1.75) | 0.001 | 1.41 (1.14-1.74) | 0.001 | 1.35 (1.10-1.67) | 0.005 | 1.34 (1.09-1.65) | 0.006 |
| High - Low | 164 | 32 (19.51) | 1.27 (0.88-1.83) | 0.200 | 1.24 (0.86-1.80) | 0.245 | 1.18 (0.82-1.71) | 0.369 | 1.16 (0.80-1.68) | 0.425 |
| High - High | 2059 | 584 (28.36) | 1.95 (1.69-2.25) | <0.001 | 1.81 (1.56-2.11) | <0.001 | 1.62 (1.38-1.90) | <0.001 | 1.56 (1.33-1.84) | <0.001 |

Model 1: unadjusted

Model 2: adjusted for age, gender, residence, marital status, educational level, ln(PCE)

Model 3: model 2 + further adjusted for smoking, alcohol consumption, hypertension, diabetes, lung disease, liver disease, digestive disease

Model 4: model 3 + further adjusted for TC, LDL, HbA1c, Scr, BUN

CI confidence interval, CVAI Chinese visceral adiposity index, PCE per capita expenditures, TC total cholesterol, LDL low-density lipoprotein, HbA1c glycosylated hemoglobin A1c, Scr serum creatinine, BUN blood urea nitrogen

^a^ High group: CVAI ≥ 88.42 (females), ≥ 101.80 (males); Low group: CVAI < 88.42 (females), < 101.80 (males)

^b^ Incident rate was presented as a percentage

# Table S10. The association of CVAI with cardiovascular and cerebrovascular diseases after excluding individuals with concomitant comorbidities.

| **CVAI^a^** | **Total N** | **No. of events (Incident rate^b^)** | **Model 1** |  | **Model 2** |  | **Model 3** |  | **Model 4** |  |
| --- | --- | --- | --- | --- | --- | --- | --- | --- | --- | --- |
|  |  |  | **HR (95% CI)** | ***P* value** | **HR (95% CI)** | ***P* value** | **HR (95% CI)** | ***P* value** | **HR (95% CI)** | ***P* value** |
| Continues |  |  |  |  |  |  |  |  |  |  |
| Per SD increase | 3328 | 541 (16.26) | 1.30 (1.19-1.43) | <0.001 | 1.27 (1.16-1.40) | <0.001 | 1.28 (1.16-1.40) | <0.001 | 1.27 (1.15-1.40) | <0.001 |
| Baseline patterns |  |  |  |  |  |  |  |  |  |  |
| Low | 1946 | 250 (12.85) | Ref. |  | Ref. |  | Ref. |  | Ref. |  |
| High | 1382 | 291 (21.06) | 1.71 (1.44-2.02) | <0.001 | 1.59 (1.33-1.90) | <0.001 | 1.60 (1.34-1.91) | <0.001 | 1.58 (1.32-1.89) | <0.001 |
| Transition patterns |  |  |  |  |  |  |  |  |  |  |
| Low - Low | 945 | 117 (12.38) | Ref. |  | Ref. |  | Ref. |  | Ref. |  |
| Low - High | 338 | 64 (18.93) | 1.57 (1.15-2.12) | 0.004 | 1.55 (1.14-2.12) | 0.005 | 1.55 (1.14-2.12) | 0.005 | 1.51 (1.11-2.07) | 0.009 |
| High - Low | 76 | 15 (19.74) | 1.68 (0.98-2.88) | 0.057 | 1.64 (0.95-2.81) | 0.073 | 1.63 (0.95-2.81) | 0.075 | 1.55 (0.90-2.67) | 0.112 |
| High - High | 847 | 205 (24.2) | 2.03 (1.62-2.55) | <0.001 | 1.92 (1.51-2.44) | <0.001 | 1.92 (1.51-2.45) | <0.001 | 1.88 (1.47-2.39) | <0.001 |

Model 1: unadjusted

Model 2: adjusted for age, gender, residence, marital status, educational level, ln(PCE)

Model 3: model 2 + further adjusted for smoking, alcohol consumption

Model 4: model 3 + further adjusted for TC, LDL, HbA1c, Scr, BUN

CI confidence interval, CVAI Chinese visceral adiposity index, PCE per capita expenditures, TC total cholesterol, LDL low-density lipoprotein, HbA1c glycosylated hemoglobin A1c, Scr serum creatinine, BUN blood urea nitrogen

^a^ High group: CVAI ≥ 88.42 (females), ≥ 101.80 (males); Low group: CVAI < 88.42 (females), < 101.80 (males)

^b^ Incident rate was presented as a percentage

# Table S11. The association of CVAI with cardiovascular and cerebrovascular diseases after excluding individuals with cancer in wave 2011.

| **CVAI^a^** | **Total N** | **No. of events (Incident rate^b^)** | **Model 1** |  | **Model 2** |  | **Model 3** |  | **Model 4** |  |
| --- | --- | --- | --- | --- | --- | --- | --- | --- | --- | --- |
|  |  |  | **HR (95% CI)** | ***P* value** | **HR (95% CI)** | ***P* value** | **HR (95% CI)** | ***P* value** | **HR (95% CI)** | ***P* value** |
| Continues |  |  |  |  |  |  |  |  |  |  |
| Per SD increase | 7631 | 1745 (22.87) | 1.32 (1.26-1.38) | <0.001 | 1.28 (1.22-1.34) | <0.001 | 1.22 (1.15-1.28) | <0.001 | 1.20 (1.14-1.27) | <0.001 |
| Baseline patterns |  |  |  |  |  |  |  |  |  |  |
| Low | 3950 | 706 (17.87) | Ref. |  | Ref. |  | Ref. |  | Ref. |  |
| High | 3681 | 1039 (28.23) | 1.67 (1.52-1.84) | <0.001 | 1.53 (1.38-1.69) | <0.001 | 1.41 (1.27-1.57) | <0.001 | 1.38 (1.24-1.54) | <0.001 |
| Transition patterns |  |  |  |  |  |  |  |  |  |  |
| Low - Low | 1842 | 332 (18.02) | Ref. |  | Ref. |  | Ref. |  | Ref. |  |
| Low - High | 684 | 160 (23.39) | 1.33 (1.10-1.60) | 0.003 | 1.30 (1.07-1.57) | 0.007 | 1.24 (1.03-1.51) | 0.026 | 1.23 (1.02-1.49) | 0.033 |
| High - Low | 182 | 46 (25.27) | 1.47 (1.08-2.01) | 0.014 | 1.43 (1.05-1.94) | 0.024 | 1.38 (1.01-1.88) | 0.044 | 1.35 (0.99-1.84) | 0.057 |
| High - High | 2234 | 713 (31.92) | 1.90 (1.67-2.16) | <0.001 | 1.74 (1.52-2.00) | <0.001 | 1.56 (1.35-1.80) | <0.001 | 1.52 (1.31-1.75) | <0.001 |

Model 1: unadjusted

Model 2: adjusted for age, gender, residence, marital status, educational level, ln(PCE)

Model 3: model 2 + further adjusted for smoking, alcohol consumption, hypertension, diabetes, lung disease, liver disease, digestive disease

Model 4: model 3 + further adjusted for TC, LDL, HbA1c, Scr, BUN

CI confidence interval, CVAI Chinese visceral adiposity index, PCE per capita expenditures, TC total cholesterol, LDL low-density lipoprotein, HbA1c glycosylated hemoglobin A1c, Scr serum creatinine, BUN blood urea nitrogen

^a^ High group: CVAI ≥ 88.42 (females), ≥ 101.80 (males); Low group: CVAI < 88.42 (females), < 101.80 (males)

^b^ Incident rate was presented as a percentage

# Table S12. Predictive performance of abdominal obesity indices for incident Cardiovascular and Cerebrovascular Diseases.

| **Variables** | **AUC (95% CI)** | ***P* for comparison** |
| --- | --- | --- |
| CVAI | 0.5897(0.5742-0.6055) | Ref. |
| VAI | 0.5528(0.5381-0.5682) | <0.001 |
| LAP | 0.5718(0.5582-0.5869) | <0.001 |
| WC | 0.5718(0.5563-0.5887) | <0.001 |
| BMI | 0.5712(0.5558-0.5871) | <0.001 |

AUC, area under the receiver operating characteristic curve; CI, confidence interval; CVAI, Chinese visceral adiposity index; VAI, visceral adiposity index; LAP, lipid accumulation product; WC, waist circumference; BMI body mass index;
